# Nanocrystalline Soft Magnetic Iron-Based Materials from Liquid State to Ready Product

**DOI:** 10.3390/nano11010108

**Published:** 2021-01-05

**Authors:** Vladimir S. Tsepelev, Yuri N. Starodubtsev

**Affiliations:** 1 Research Center for Physics of Metal Liquids, Institute of New Materials and Technologies, Ural Federal University, Mira Str.19, 620002 Ekaterinburg, Russia; yunstar@mail.ru; 2 Gammamet Research and Production Enterprise, Tatishchev Str. 92, 620028 Ekaterinburg, Russia

**Keywords:** soft magnetic materials, nanocrystalline structure, liquid multicomponent alloy, grain growth inhibitor, magnetic hysteresis, magnetic core, core losses, transformer, inductive component

## Abstract

The review is devoted to the analysis of physical processes occurring at different stages of production and application of nanocrystalline soft magnetic materials based on Fe–Si–B doped with various chemical elements. The temperature dependences of the kinematic viscosity showed that above a critical temperature, the viscosity of multicomponent melts at the cooling stage does not coincide with the viscosity at the heating stage. Above the critical temperature, the structure of the melt is more homogeneous, the amorphous precursor from such a melt has greater plasticity and enthalpy of crystallization and, after nanocrystallization, the material has a higher permeability. The most effective inhibitor elements are insoluble in α-Fe and form a smoothed peak of heat release during crystallization. On the other hand, the finest nanograins and the highest permeability are achieved at a narrow high-temperature peak of heat release. The cluster magnetic structure of a nanocrystalline material is the cause of magnetic inhomogeneity, which affects the shape of the magnetic hysteresis loop and core losses.

## 1. Introduction

Nanocrystalline soft magnetic materials were discovered by Yoshizawa, Yamauchi and Oguma in 1986 [1,2]. This discovery was preceded by numerous research and development projects concerning amorphous soft magnetic materials. To obtain an amorphous structure, rapid quenching of the metal melt with a cooling rate of about 10^6^ K·s^−1^ is used. Under industrial conditions, an amorphous ribbon 20 μm thick is produced using the planar flow casting process [3]. Amorphous Fe–B alloy, in which boron contributes to amorphization, is widely used in scientific research. For industrial purposes, the Fe–Si–B alloy [4] is more suitable, in which silicon contributes to an increase in the crystallization temperature and a decrease in the coercive force.

Crystallization of the amorphous Fe–Si–B alloy leads to the release of heat and an increase in the density, while the crystallites grow to 0.1–1 μm [5]. The first crystals are nucleated on the surface of the ribbon [6], and then the crystallization front propagates deep into the material [7]. As the grain size decreases, the length of grain boundaries increases. The grain boundaries are crystal lattice defects. Therefore, an increase in the defect density is accompanied by an increase in the coercive force of the magnetic material [8]. When the grain size decreases to 0.1 μm, the coercive force can reach several kA·m^−1^. In the Fe–Si–B alloy, Yoshizawa and coworkers additionally introduced Cu, which has the effect of nucleation, and Nb, which increases the crystallization temperature. As a result, the Fe–Cu–Nb–Si–B alloy was obtained, which after crystallization had a crystal size significantly less than 100 nm, a coercive force of less than 1 A·m^−1^ and an initial permeability of about 100,000 [2]. This alloy with ultrafine grain structure has been named Finemet.

A decrease in the coercive force *H_c_* in a nanocrystalline soft magnetic material is associated with a weakening of the magnetic anisotropy due to interaction of nanocrystals [9,10]. It should be taken into account that *H_c_* is proportional to the magnetic anisotropy constant *K*, and the permeability μ is inversely proportional to *K* [11]. In a polycrystalline material, the macroscopic magnetic anisotropy coincides with the local magnetic anisotropy in each individual crystallite. This relationship is violated if the grain size is less than the magnetocrystalline exchange length:

(1) $$L_{0}\approx \sqrt{\frac{A}{\left|K_{1}\right|}},$$

where *A* is the exchange constant, J·m^−1^, *K*_1_ is the first constant of magnetic crystallographic anisotropy, J·m^−3^. The quantity *L*_0_ compares the exchange energy with the energy of magnetic crystallographic anisotropy [12] and determines the propagation of a magnetic inhomogeneity of the domain wall type, since this quantity also determines the width of the 180° Bloch domain wall.

In the model of random magnetic anisotropy [13], the material consists of structural regions (in our case, grains) with size *d*, having the same exchange constant *A*, magnetic crystallographic anisotropy constant *K*_1_, and saturation magnetization *M_s_*; however, the direction of the easy magnetization axes has random distribution. A low energy of magnetic anisotropy is achieved if the magnetization in each region is oriented along the local axis of easy magnetization. However, a sharp change in the orientation of the magnetization in neighboring regions creates a large exchange energy. Therefore, within a certain length *L_ex_*, which is much larger than the structural regions *d*, such an interconnected change in the directions of magnetization should occur, which will correspond to the minimum magnetic energy. The *L_ex_* characterizes the size of the region within which the magnetizations of the structural regions correlate or interact with each other, i.e., the structural regions are connected with each other by some exchange interaction. For this reason, *L_ex_* is called the exchange correlation length.

Since *L_ex_* >> *d*, the fluctuations of magnetization are averaged over a large number of regions with different directions of the easy axes. In this case, in the region of volume (*L_ex_*)^3^, there is always an axis of the easiest magnetization, which is determined by statistical fluctuations. It follows from statistical considerations that the magnetic anisotropy constant 〈*K*〉 is less than the maximum value *K*_1_ by the square root of the number of independent contributions. Thus, the effective magnetic anisotropy constant 〈*K*〉 in the exchange correlation region (*L_ex_*)^3^ can be written in the form [14]:

(2) $$\langle K\rangle =\frac{K_{1}}{\sqrt{N}},$$

where *N* is the number of structural regions in the volume of exchange correlation (*L_ex_*)^3^. Considering that,

(3) $$N={\left(\frac{L_{ex}}{d}\right)}^{3},$$

we obtain,

(4) $$\;K\;=K_{1}{\left(\frac{d}{L_{ex}}\right)}^{\frac{3}{2}}.$$

**Figure 1:**
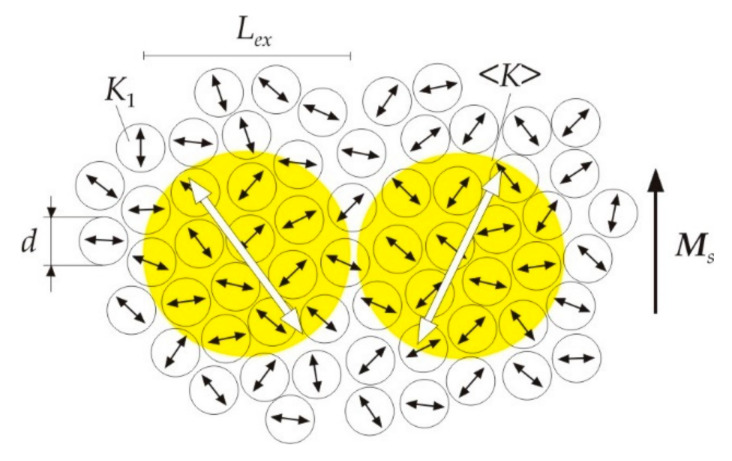
Schematic representation of the magnetic structure of a nanocrystalline soft magnetic alloy. Adapted from [15].

From a magnetic point of view, a nanocrystalline magnetic material consists of exchange correlation regions or ferromagnetic clusters. Figure 1 schematically shows the magnetic structure of a nanocrystalline alloy [15]. Individual crystal grains of size *d* with the magnetic anisotropy constant *K*_1_ have random directed easy magnetization axes, indicated by black arrows. Ferromagnetic clusters have the size *L_ex_*, the effective magnetic anisotropy constant 〈*K*〉, and the easiest magnetization axes, indicated by double arrows.

The exchange correlation length *L_ex_* can be found from the condition for the minimum magnetic energy density of the ferromagnet, which is the sum of the magnetic anisotropy energy density [13]:

(5) $$E_{ma}=-\langle K\rangle$$

and exchange energy density,

(6) $$E_{ex}\approx \frac{A}{L_{ex}^{2}}.$$

From (4)–(6) it follows that magnetic energy of a ferromagnet is minimal at:

(7) $$L_{ex}\approx \frac{A^{2}}{K_{1}^{2}d^{3}}.$$

After substituting *L_ex_* in (4), we obtain the effective magnetic anisotropy constant 〈*K*〉 in the form:

(8) $$\langle K\rangle =\frac{K_{1}^{4}d^{6}}{A^{3}}=K_{1}{\left(\frac{d}{L_{0}}\right)}^{6}.$$

Thus, the effective magnetic anisotropy constant 〈*K*〉 depends on the grain size as *d*^6^. The exponent approaches 3 in the presence of a strong induced magnetic anisotropy [16] or in the presence of a grain size distribution [17,18].

**Figure 2:**
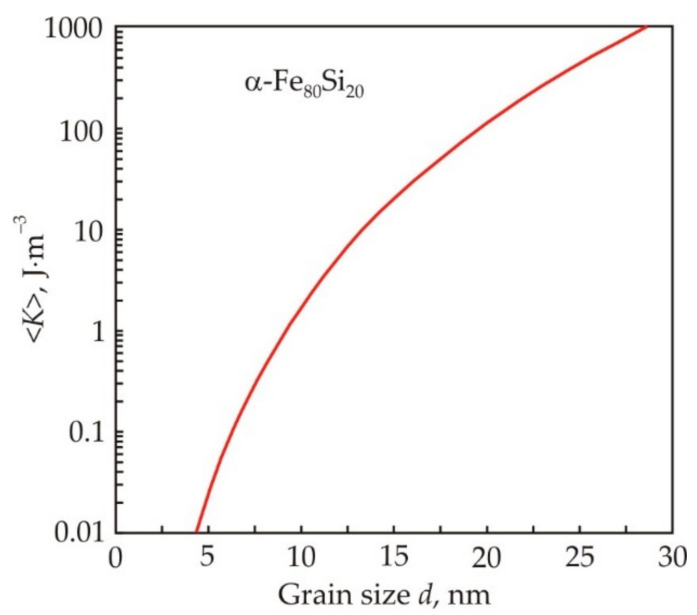
Dependence of the effective magnetic anisotropy constant 〈*K*〉 on the grain size *d* in the α-Fe_80_Si_20_ alloy, obtained in the random anisotropy model. Reproduced with permission from [19] (pp. 240–244).

In a nanocrystalline alloy, the structural region is a crystal grain. For nanocrystals of the Fe_80_Si_20_ solid solution, the magnetic anisotropy constant *K*_1_ is 8 × 10^3^ J·m^−3^ and the exchange constant *A* is 10^−11^ J·m^−1^. After substituting these values in (1), we obtain the magnetocrystalline exchange length *L*_0_ = 35 nm. Figure 2 shows the dependence of the effective magnetic anisotropy constant 〈*K*〉 on the grain size *d* for *L*_0_ = 35·nm in accordance with formula (8) [19] (pp. 240–244). At a grain size of 10 nm, the effective constant 〈*K*〉 decreases by about 2000 times as compared with the magnetic crystallographic constant *K*_1_.

From formula (7) for a grain size *d* = 10 nm, we calculate the size of the ferromagnetic cluster *L_ex_*, which is of the order of 1 μm. Thus, about 100 nanograins are located along the diameter of the α-Fe_80_Si_20_ ferromagnetic cluster. From formulas (7) and (8), we obtain the following expression for the size of a ferromagnetic cluster:

(9) $$L_{ex}\approx \sqrt{\frac{A}{\langle K\rangle }}.$$

Consequently, with a decrease in the effective magnetic anisotropy constant 〈*K*〉, the size of ferromagnetic clusters *L_ex_* will increase.

The production of magnetic systems from nanocrystalline materials can be divided into several technological operations [19] (pp. 244–249). The initial operation is the smelting of an alloy with a given chemical composition. The next step is rapid quenching of the melt, resulting in an amorphous precursor in the form of a 20 μm thick ribbon. Heat treatment of the amorphous precursor ensures the formation of a nanocrystalline structure with a guaranteed level of magnetic properties. As a rule, magnetic circuits or cores are first made from an amorphous precursor, and then they are subjected to heat treatment. Heat is released during crystallization, so the optimal heat treatment depends not only on the chemical composition of the alloy, but also on the size of the cores.

The purpose of this review is to analyze the physical processes occurring at different stages of the production and application of nanocrystalline soft magnetic materials. The object was Fe–Si–B alloys doped with various chemical elements, which have found wide practical application. The section “Multicomponent melts” analyzes the effect of temperature and chemical composition of melts on the structure of an amorphous precursor and nanocrystalline alloy. The section “Nanocrystallization” discusses the main features of nanocrystallization, and “Grain growth inhibitors” evaluates the effectiveness of using inhibitors to achieve the optimal nanocrystalline structure. The section “Solute elements in α-Fe” analyzes the effect of Ni, Co, Si, Al soluble in nanocrystals on magnetic properties. The “Core losses” section contains an analysis of magnetic losses, which play a critical role in various applications of soft magnetic materials. The last section compares the magnetic properties of various nanocrystalline soft magnetic materials and analyzes their application in power electronics.

## 2. Multicomponent Melts

Nanocrystalline soft magnetic materials are multicomponent alloys, for example, Finemet has the composition Fe_73.5_Cu_1_Nb_3_Si_13.5_B_9_ [2]. In Fe–Si–B-based alloys, there is a strong covalent bond in Fe–B and Fe–Si, both in the amorphous and in the liquid states, and the B and Si atoms interact weakly [20]. A non-uniform distribution of atoms takes place in the melt [21]. The tendency for different atoms to approach each other leads to the formation of clusters with a structure close to Fe_3_B and Fe_2_B [22,23], as well as Fe_3_Si and FeSi [24,25], and boron-based clusters are formed more easily [26]. The inhomogeneous structure of metal melts manifests itself in the features of the temperature dependences of physical properties.

Among the physical properties, viscosity most reflects the structure of melts. Kinematic viscosity ν, m^2^·s^−1^, must correspond to the equation [27]:

(10) $$\nu =\nu_{0}e^{\frac{E_{a}}{RT}},$$

where *T* is the absolute temperature, K, ν_0_ is a pre-exponential factor with the dimension of the kinematic viscosity m^2^·s^−1^, *E_a_* is the activation energy of the viscous flow, J·mol^−1^, *R* is the gas constant, J·K^−1^·mol^−1^. At constant ν_0_ and *E_a_*, the melt viscosity decreases with increasing temperature according to Equation (10). After taking the logarithm we obtain:

(11) $$\ln\nu =\ln\nu_{0}+\frac{E_{a}}{RT}.$$

Thus, the logarithm of the kinematic viscosity is a linear function of the inverse absolute temperature. This representation allows one to calculate the pre-exponential factor ν_0_ and the activation energy *E_a_* from the temperature dependence of the viscosity, in which the viscosity is presented on a logarithmic scale, and the absolute temperature is in the inverse scale.

**Figure 3:**
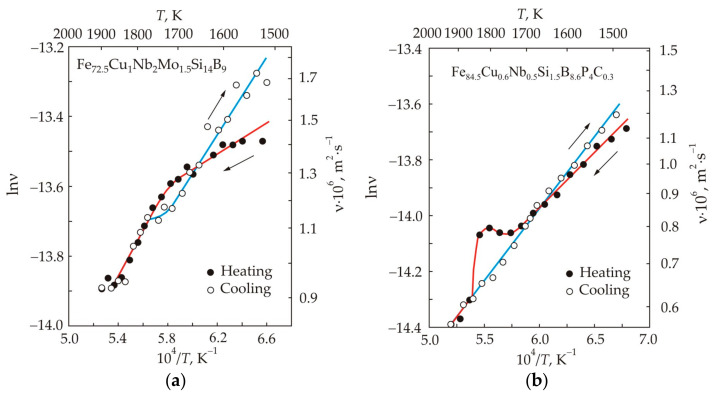
Temperature dependences of kinematic viscosity ν for nanocrystalline alloys Fe_72.5_Cu_1_Nb_2_Mo_1.5_Si_14_B_9_ (**a**) and Fe_84.5_Cu_0.6_Nb_0.5_Si_1.5_B_8.6_P_4_C_0.3_ (**b**). The arrows show the direction of temperature change.

Figure 3 shows the kinematic viscosity in logarithmic scale and absolute temperature in the inverse scale for two alloys Fe_72.5_Cu_1_Nb_2_Mo_1.5_Si_14_B_9_ (a) and Fe_84.5_Cu_0.6_Nb_0.5_Si_1.5_B_8.6_P_4_C_0.3_ (b), which have significantly different silicon contents. In accordance with Equation (11), the temperature dependences of viscosity should represent a straight line, but in multicomponent melts they have a number of characteristic features [28]. When the melt is heated above a certain temperature *T_h_*, the cooling curve coincides with the heating curve at *T* > *T_h_* and deviates at *T* < *T_h_*. In this case, there is a temperature region near *T_h_*, within which the heating and cooling curves form a hysteresis loop. For these reasons, the temperature *T_h_* can be called the hysteresis temperature or branching temperature *T_br_* [29].

The greatest nonlinearity of the temperature dependence of viscosity in logarithmic and inverse scales is observed during heating. The temperature at which the activation energy in the Fe_72.5_Cu_1_Nb_2_Mo_1.5_Si_14_B_9_ melt changes can be called the critical temperature *T_k_*, see Figure 3a. This temperature corresponds approximately to the middle of the temperature range for the hysteresis loop. Another type of anomaly manifests itself in the form of a local increase in viscosity, as for the Fe_84.5_Cu_0.6_Nb_0.5_Si_1.5_B_8.6_P_4_C_0.3_ melt in Figure 3b. The critical temperature is associated with the rearrangement of the melt structure. In particular, in the high-temperature region, the formation of a more homogeneous structure occurs due to the dissolution of clusters; therefore, this temperature is sometimes called dissolution temperature *T_d_* [29]. At the cooling stage, the non-linearity of the temperature dependence of viscosity is less pronounced or completely absent.

In binary melts Fe-Si [25] and Fe-B [30], the temperature dependences of the kinematic viscosity were obtained, which were similar to those for the Fe_72.5_Cu_1_Nb_2_Mo_1.5_Si_14_B_9_ and Fe_84.5_Cu_0.6_Nb_0.5_Si_1.5_B_8.6_P_4_C_0.3_ melts. The viscosity of the Fe-Si melt varied smoothly during heating and cooling. An anomaly was observed in the Fe-B melt, which manifested itself in a local increase in viscosity near the critical temperature. The appearance of the anomaly in boron-containing melts was associated with the rearrangement of FeB- and Fe_2_B-based clusters, which transform with increasing temperature into Fe_4_B- [30] or Fe_3_B-based clusters [31]. A local increase in viscosity near the critical temperature is not observed in the Fe_72.5_Cu_1_Nb_2_Mo_1.5_Si_14_B_9_ melt. In addition to B, this alloy also contains a large amount of Si. Apparently, in the presence of silicon, a significant part of boron is bound to less stable B–Si and Fe–B–Si compounds [32].

Long time exposure of the melt at a fixed temperature is accompanied by fluctuations in viscosity, the amplitude of which significantly exceeds the measurement error [33]. The greatest temporal instability of the melt is observed at the temperature of structural transformations [34]. The reason for the oscillations can be a transition from a non-equilibrium melt structure inherited from the initial crystalline phases to an equilibrium state, or random oscillations associated with the periodic appearance and destruction of cluster structures, or an intense structural transformation of the melt [35].

**Figure 4:**
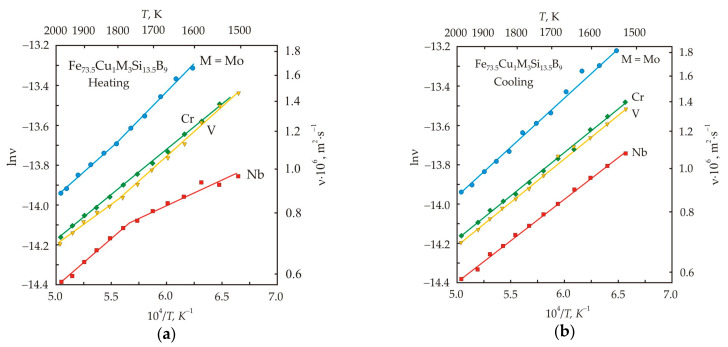
Temperature dependences of the kinematic viscosity ν for nanocrystalline Fe_73.5_Cu_1_M_3_Si_13.5_B_9_ alloys with different inhibitors M = Nb, Mo, V, Cr at the stage of heating (**a**) and cooling (**b**). Reproduced with permission from [36].

Figure 4 shows the kinematic viscosity in logarithmic scale and absolute temperature in the inverse scale for Fe_73.5_Cu_1_M_3_Si_13.5_B_9_ melts with different inhibitors M = Nb, Mo, V, Cr at the stage of heating (a) and cooling (b) [36]. At the heating stage, the temperature dependences of melts with Nb, Mo, and V have two linear sections, the slope of which changes at the critical temperature *T_k_* = 1770 K. In a melt with Nb, the activation energy increases upon going to the high-temperature region. This pattern is also observed in the Fe_72.5_Cu_1_Nb_2_Mo_1.5_Si_14_B_9_ melt, which is dominated by Nb, see Figure 3a. In melts with Mo and V, the activation energy decreases during the transition to the high-temperature region. In a melt with Cr in the entire temperature range, the dependence is a straight line. At the cooling stage, linear dependences were obtained in the entire temperature range for all melts.

Table 1 shows the calculated values of the activation energy *E_a_* for melts with various inhibitors [36]. In the low-temperature region, a melt with Nb has the lowest *E_a_* = 21 kJ·mol^−1^, and a melt with Mo has the highest one, *E_a_* = 47 kJ·mol^−1^. In the high-temperature region at the stages of heating and cooling, the activation energies of melts differ insignificantly. Since after cooling the viscosity of the melts does not recover to its initial value, it can be concluded that the melt underwent irreversible changes.

The viscosity of Fe_73.5_Cu_1_M_3_Si_13.5_B_9_ melts with various inhibitors correlates well with the free volume [36]. The Nb melt has the largest free volume, while the Mo melt has the smallest. The metallic radius of Nb, Mo, V, Cr, and Fe is 146, 139, 134, 128, and 126 pm, respectively [37]. At a temperature of 1000 K, Nb is slightly soluble in α-Fe, and the solubility of other elements increases with decreasing atomic size. Atoms of inhibitory elements occupy vacant positions in a substitutional solid solution. A large atomic radius favors the formation of vacancies [38], which are concentrated near inhibitory atoms with the formation of vacancy clusters [39]. Consequently, an alloy containing Nb should have the highest concentration of vacancies already before the onset of melting. A high concentration of vacancies should facilitate easier activation of the viscous flow.

At the stage of melting, the melt retains the short-range order structure inherited from the multiphase solid state. In this case, the structural component of the melt is clusters, the size of which depends on the temperature [40]. As the temperature increases, the cluster size decreases, and at temperatures above *T_k_*, the melt structure becomes more homogeneous. If the melt is heated above the critical temperature and then quickly transferred to an amorphous solid state, then it can be expected that the homogeneous structure of the liquid will remain in the amorphous state. The heredity of the structure was investigated on samples of amorphous ribbon, which was obtained after heating the melt above and below the critical temperature. After high-temperature holding of melt, an amorphous ribbon has a lower ordering of atoms [41], a larger molar volume [42], greater plasticity [41,43], hardness and fracture toughness [43], and a higher enthalpy of crystallization [42,44]. Thus, the heredity of the melt structure remains in the amorphous solid state after melt quenching.

For the study of melts, an ingot is usually used, which was obtained after melting the alloy and slow cooling. The melt viscosity depends on its prehistory. Therefore, it should be expected that the structure and properties of the melt from the amorphous precursor will be different. When choosing the temperature regime, it was taken into account that the Fe_72,5_Cu_1_Nb_2_Mo_1,5_Si_14_B_9_ alloy has a critical temperature of 1760 K. The melt was heated to 1795 K, i.e., above the critical temperature, kept at this temperature for 5 min, then cooled to 1750 K, held for another 5 min and then the melt was quickly quenched. This mode of obtaining an amorphous precursor and a melt from this amorphous ribbon will be called “overheated”. In the “not overheated” mode, the melt was heated to 1750 K, i.e., below the critical temperature, kept at this temperature for 5 min, and then the melt was quickly quenched.

**Figure 5:**
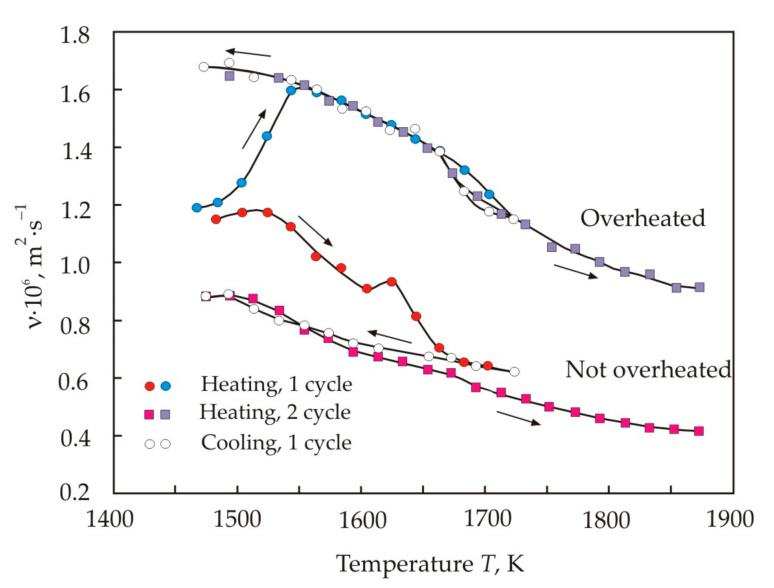
Temperature dependences of kinematic viscosity ν for the first heating–cooling cycle and for the second heating in the overheated and not overheated melts prepared from the amorphous Fe_72.5_Cu_1_Nb_2_Mo_1.5_Si_14_B_9_ ribbon. The arrows show the direction of temperature change [45].

Figure 5 shows the temperature dependences of the kinematic viscosity at the first heating–cooling cycle and at the second heating of the melt obtained from the amorphous Fe_72,5_Cu_1_Nb_2_Mo_1,5_Si_14_B_9_ ribbon [45]. It follows from the figure that immediately after the melting of the amorphous ribbon, the viscosity of the overheated and not overheated melts is almost the same. However, after increasing the temperature to about 1540 K, the curves diverge significantly. The viscosity of the overheated melt goes up sharply, while the viscosity of the not overheated melt changes insignificantly. At temperatures above 1540 K, the viscosity decreases in accordance with the classical concept of the free volume [46,47].

The temperature curve of the viscosity of the overheated melt reaches a stable trajectory already at the second heating, with the exception of a small loop in the range of 1670–1720 K. In the not overheated melt, the trajectory of viscosity continued to decrease. From Figure 5, we can conclude that the melt obtained from the amorphous ribbon is non-equilibrium, and the not overheated melt has the greatest deviation from the equilibrium structure. The difference in the viscosity indicates a different structure of atomic groups participating in the viscous flow.

**Figure 6:**
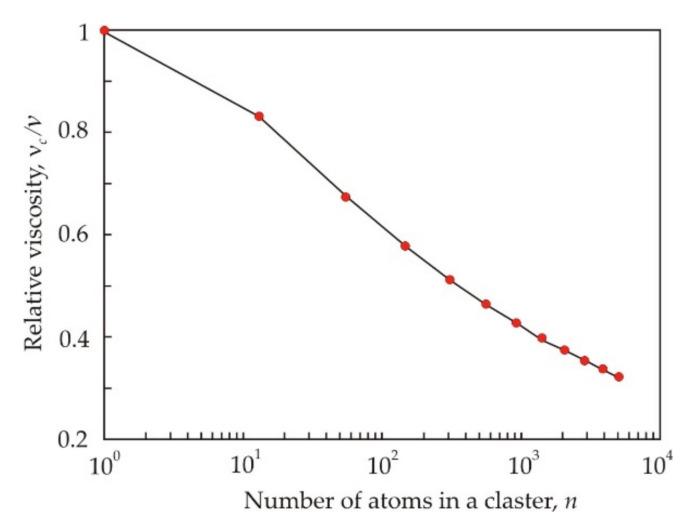
The dependence of relative melt viscosity ν*_c_*/ν on the number of atoms in a cluster *n* [45].

In the hard spheres model, the viscosity of the melt can be represented as [48]:

(12) $$\nu \propto a\sqrt{\frac{k_{B}T}{m}},$$

where *a* is the atomic size, m, *m* is the atomic mass, kg, *k_B_* = 1.38 × 10−23 J·K^−1^ is Boltzmann constant. If clusters are involved in a viscous flow, then it should be taken into account that the cluster mass is proportional to the number of atoms in the cluster, and the cluster size is not proportional [49]. Figure 6 shows the dependence of the relative viscosity of the melt νc/ν, where νc is the viscosity of a liquid consisting of clusters on the number of atoms in the cluster n [45]. It follows from the Figure that the viscosity of the liquid should decrease with increasing cluster size. The same conclusion can be obtained on the basis of general concepts [28], since as the cluster size decreases, the energy of interaction between them increases, and this should lead to an increase in the viscosity.

When the amorphous ribbon is heated, crystallization and melting processes take place. At the first stage of crystallization, nanocrystalline grains are formed with an average size of about 10 nm [50]. It is important that in an amorphous ribbon prepared from an overheated melt, there are noticeably more small grains 1–2 nm in size [44]. At the second stage of crystallization, metastable phases based on Fe–B and Fe–Si are formed [51], while Fe–B, Fe–Nb, and Nb–B clusters dominate in the melt [52]. Apparently, the finer nanocrystalline structure in the superheated ribbon became the basis for the formation of a finely dispersed melt structure. This makes it possible to associate the increased viscosity of the superheated melt with the small size of atomic groups in the liquid.

**Figure 7:**
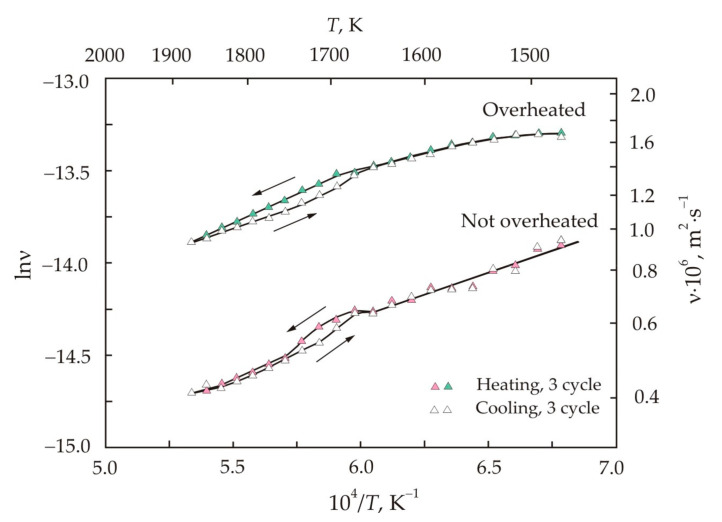
Temperature dependences of kinematic viscosity ν for the third heating—cooling cycle in the overheated and not overheated melt prepared from the amorphous Fe_72.5_Cu_1_Nb_2_Mo_1.5_Si_14_B_9_ ribbon. The arrows show the direction of temperature change [45].

Figure 7 shows that in the third heating–cooling cycle, a good stabilization of the trajectories of the viscosity curves was achieved [45]. Despite this, in the region of the critical temperature, irreversibility remains, and it manifests itself in the form of a hysteresis loop. The branching of the curves begins at a temperature of 1670 K. Moreover, in this region there is also an increased slope of the curves, which indicates an increase in the activation energy of the viscous flow. The calculation results for the activation energy *E_a_* and pre-exponential factor ν_0_ are presented in Table 2 [45]. The entire temperature range was divided into intervals with different activation energies. The calculated values were obtained by linear extrapolation of the experimental points for each temperature range.

Table 2 also shows the calculated cluster sizes *d*. The calculation was carried out using the Eyring model based on the transition state theory [47]. In the Eyring model, the viscosity can be represented as [53]:

(13) $$\nu ={\left(\frac{2\pi k_{B}T}{\rho }\right)}^{\frac{1}{2}}{\left(\frac{v_{f}}{v}\right)}^{\frac{1}{3}}d^{\frac{-1}{2}}e^{\frac{E_{a}}{RT}},$$

where ρ is the melt density, kg·m^−3^, *d* is the particle diameter, m, v is the volume occupied by one particle, m^3^, v*_f_* is the free volume of liquid, m^3^, v*_f_*/v is the relative free volume. From formula (13), it follows that the melt viscosity decreases with increasing particle size. Comparing Equations (10) and (13), we obtain:

(14) $$d=\left(\frac{2\pi k_{B}T}{\rho }\right){\left(\frac{v_{f}}{v}\right)}^{\frac{2}{3}}\nu_{0}^{-2}.$$

To calculate the cluster size, we used the measured density and free volume of melts [36,53].

When discussing the data on the cluster size, it is necessary to take into account that the model concepts differ from a real multicomponent liquid, in which atoms and clusters of different compositions and sizes coexist. For the low-temperature region in the overheated regime, the effective cluster size of 0.05 nm was obtained, which can be taken as the atomic size, taking into account the accuracy of model representations. Thus, in the low-temperature region, the viscosity of the melt is associated with the vibrations of individual atoms, which are mainly located near their equilibrium positions. With increasing temperature, the mobility of atoms increases, but strong bonds Fe–B, Fe–Nb(Mo), and Nb(Mo)–B restrain decomposition into individual atoms and ensure the formation of clusters.

**Figure 8:**
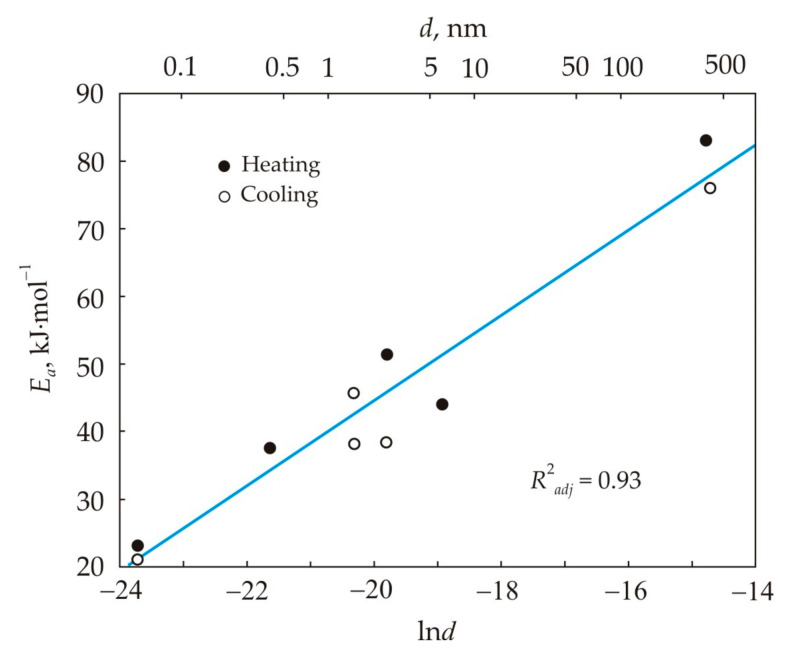
Relationship between the activation energy of viscous flow *E_a_* and the cluster size *d*.

Table 2 shows that the cluster size is higher in the not overheated melt, which also has a lower viscosity. The largest cluster size falls on the region of the hysteresis loop, where the activation energy is also the highest. Table 2 also shows that the cluster size and activation energy are related. The dependence of the activation energy *E_a_* on the cluster size *d* is shown in Figure 8. This relationship can be written as a linear function:

(15) $$E_{a}=a+b\ln d,$$

which has an adjusted coefficient of determination *R*^2^*_adj_* = 0.93. In formula (15), *a* = 170,000 J·mol^−1^ and *b* = 6300 J·mol^−1^, and the activation energy and the cluster size have the dimensions J·mol^−1^ and m, respectively. The quantity *b* characterizes the change in the activation energy per unit cluster size on a logarithmic scale. formula (15) can be transformed to the form:

(16) $$d=1.76\times 10^{-12}e^{\frac{E_{a}}{b}},$$

which expresses the dependence of the cluster size on the activation energy.

The study of multicomponent melts shows that the structures of the liquid and solid states are interrelated. The melt heated above the critical temperature has the most homogeneous structure. An amorphous precursor obtained from a homogeneous melt has high plasticity and hardness, as well as the enthalpy of crystallization. After nanocrystallization of an amorphous precursor from a overheated melt, a magnetic material with a higher permeability was obtained [44].

## 3. Nanocrystallization

The main stage in the production of nanocrystalline soft magnetic materials is the heat treatment of the amorphous precursor, as a result of which a nanocrystalline structure is formed. The iron-based alloy Fe_73.5_Cu_1_Nb_3_Si_13.5_B_9_, which is associated with the Finemet brand, was the first nanocrystalline soft magnetic material with high permeability. At present, this alloy with slight variations in chemical composition is the basis for the production of nanocrystalline soft magnetic materials. At the initial stage of heat treatment, copper-rich clusters are formed, which create a chemical inhomogeneity of the amorphous matrix. These inhomogeneities are centers of heterogeneous nucleation of a large number of α-FeSi crystals throughout the material volume [54,55,56]. Niobium inhibits the growth of the crystalline phase to a higher temperature and also prevents the formation of iron borides [57,58].

**Figure 9:**
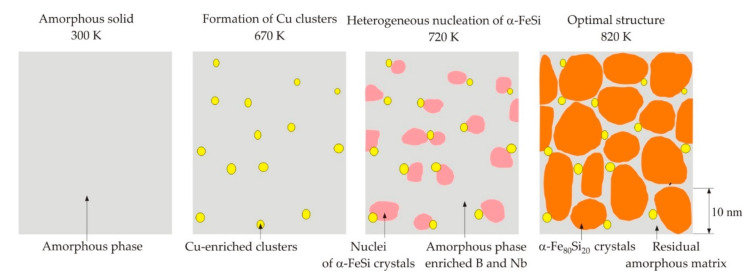
Different stages of the formation of a nanocrystalline structure in the Fe_73.5_Cu_1_Nb_3_Si_13.5_B_9_ alloy. Adapted from [59].

Figure 9 schematically shows the different stages of nanocrystallization of the Finemet alloy [59]. At the stage of heterogeneous nucleation, crystals of the α-FeSi solid solution are formed, in which the silicon content is lower than its nominal value in the amorphous state [59]. With time, silicon diffuses into the crystalline phase and the silicon content in the solid solution increases to 20 at. % [59,60]. At the final stage of nanocrystallization, grains are formed, the composition of which is close to Fe_80_Si_20_ and Fe_3_Si, surrounded by a residual amorphous matrix. The volume fraction of the crystalline phase is about 0.7 [15].

**Figure 10:**
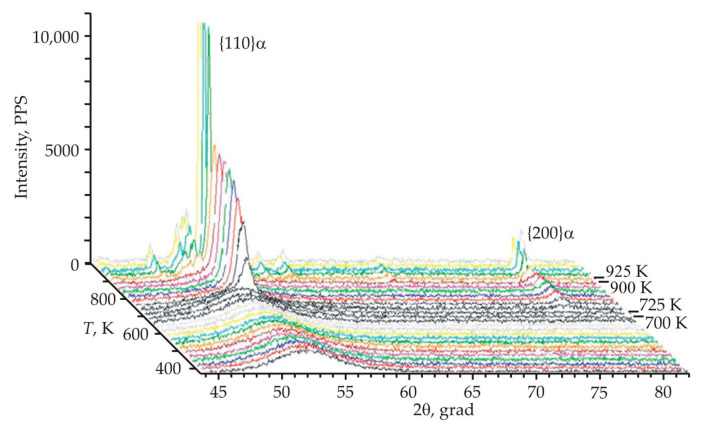
Diffraction patterns of the Fe_72.5_Cu_1_Nb_2_Mo_1.5_Si_14_B_9_ alloy during heating [61].

Figure 10 shows the diffraction patterns of the Fe_72.5_Cu_1_Nb_2_Mo_1.5_Si_14_B_9_ alloy during heating with a step of 25 K [61]. At temperatures below 725 K, the diffraction patterns have the form of halos, which correspond to the amorphous state. The first appearance of a small reflection at a larger angle 2θ coincides with the onset of crystallization at a temperature of 725 K. With increasing temperature, the halo is replaced by a set of individual reflections {110}, {200}, which correspond to the bcc lattice.

At a temperature of 880 K, boride and intermetallic phases are found. A sharp increase in the height of the peaks with a simultaneous decrease in their width was recorded at a temperature of 925 K. This indicates a sharp increase in the degree of perfection of the crystal lattice, which can be associated with recrystallization and grain coarsening. An intense release of energy 386 and 88 kJ·mol^−1^ occurs at temperatures of 815 and 950 K and this corresponds to the peaks of crystallization and recrystallization.

**Figure 11:**
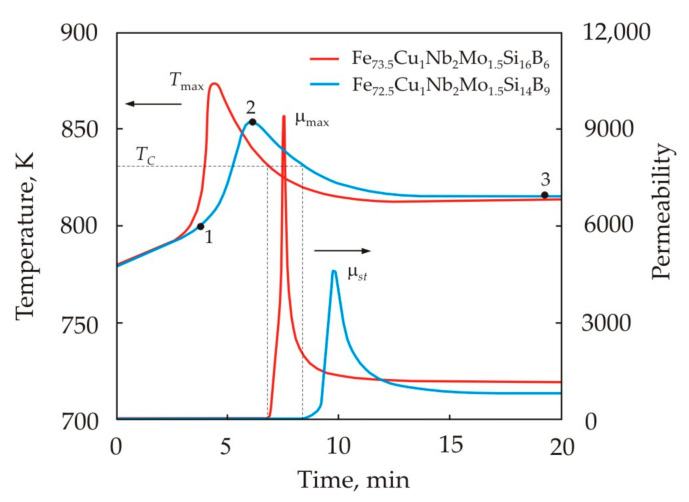
The change in time of the temperature and permeability of the cores made of Fe_72.5_Cu_1_Nb_2_Mo_1.5_Si_14_B_9_ and Fe_73.5_Cu_1_Nb_2_Mo_1.5_Si_16_B_6_ alloys during nanocrystallization in a furnace with a constant temperature of 813 K. Adapted from [50].

Figure 11 shows the change in time of the temperature and permeability of the cores made of Fe_72.5_Cu_1_Nb_2_Mo_1.5_Si_14_B_9_ and Fe_73.5_Cu_1_Nb_2_Mo_1.5_Si_16_B_6_ alloys during nanocrystallization in a furnace with a constant temperature of 813 K [50]. To fix the structure at different stages of crystallization, the cores were taken out of the furnace and quickly cooled in the time indicated by the dots in Figure 11. The peak temperature in the core indicates the release of heat during the crystallization process. The higher Si content in the Fe_73.5_Cu_1_Nb_2_Mo_1.5_Si_16_B_6_ alloy increases the enthalpy of crystallization [62]. Therefore, this alloy has a higher heating rate and the peak temperature *T*_max_.

The peak of permeability coincides with the formation of a ferromagnetic state in nanocrystals at the cooling stage. Figure 11 shows that the Curie temperature of the primary crystalline phase is the same for alloys with different silicon contents. After reaching the peak value µ_max_, the permeability decreases rather quickly, stabilizing at the level of µ*_st_* [50,63]. By that time, the diffusion of chemical elements, as well as the residual movement of grain boundaries, still continues. Near the Curie point, the magnetic anisotropy constant of the crystalline phase is low. Therefore, the permeability is high enough, despite the fact that the crystals are surrounded by a non-magnetic amorphous matrix. Due to the diffusion of silicon, the chemical composition of the crystalline and amorphous phases changes over time. The stabilization of the chemical composition, as well as the occupation of stable positions by the diffusing atoms at the sites of the crystal lattice, leads to a decrease and stabilization of the permeability.

The structure of sample 1 in Figure 11 represents an amorphous matrix with a small fraction of grains, the average size of which is 6 nm [50]. The maximum frequency distribution falls on grains 2 nm in size, which can be identified as nuclei of the crystalline phase. The structures of samples 2 and 3 practically do not differ with an average grain size of 10 nm. After cooling, sample 1 had an initial permeability μ_0.08_ = 5600 and a coercive force *H_c_* = 1.25 A/m, while in samples 2 and 3 the magnetic properties were the same, μ_0.08_ = 70,000 и *H_c_* = 0.50 A/m.

**Figure 12:**
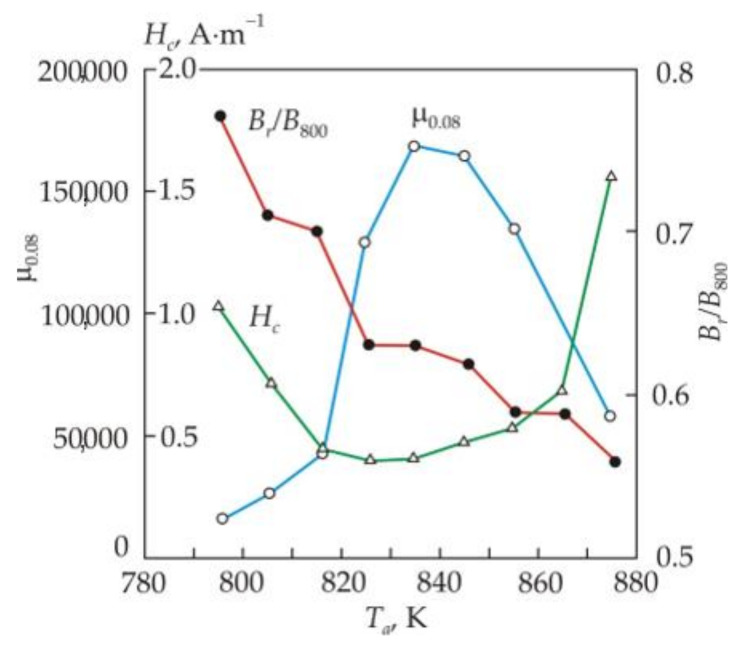
Dependences of the initial permeability μ_0.08_, coercive force *H_c_*, and remanence ratio *B_r_*/*B*_800_ on the annealing temperature *T_a_* of the Fe_72.5_Cu_1_Nb_2_Mo_1.5_Si_14_B_9_ alloy. Adapted from [64].

Figure 12 shows the dependences of the initial permeability μ_0.08_, the coercive force *H_c_* and the remanence ratio *B_r_*/*B*_800_ on the annealing temperature *T_a_* of the Fe_72.5_Cu_1_Nb_2_Mo_1.5_Si_14_B_9_ alloy being held at a fixed temperature for 1 h [64]. It follows from the Figure that the remanence ratio decreases almost linearly with increasing temperature, and the initial permeability μ_0.08_ and the coercive force *H_c_* have an optimal temperature range. To obtain a high initial permeability, the optimum annealing temperature should be approximately 20 K higher than the minimum coercive force. High or low remanence ratio *B_r_*/*B*_800_ distinguishes low-temperature and high-temperature annealing of nanocrystalline soft magnetic alloy.

**Figure 13:**
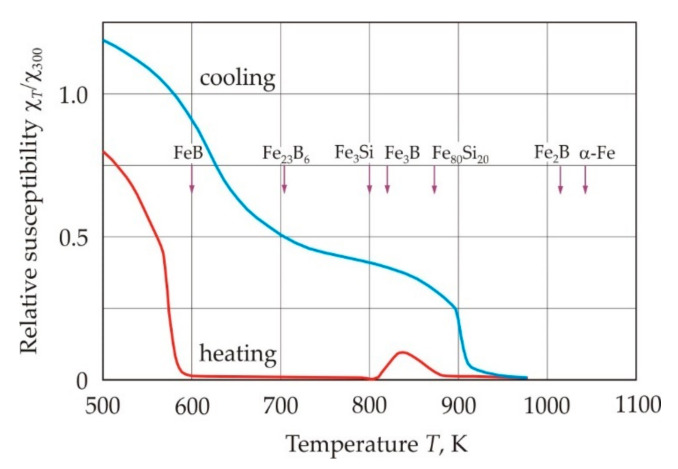
Temperature dependence of the relative magnetic susceptibility χ*_T_*/χ_300_ of a ribbon made of the Fe_72.5_Cu_1_Nb_2_Mo_1.5_Si_14_B_9_ alloy during heating and cooling and the Curie point of crystalline phases. Reproduced with permission from [65].

Figure 13 shows the dependences of the relative magnetic susceptibility χ*_T_*/χ_300_ of a ribbon made of the Fe_72.5_Cu_1_Nb_2_Mo_1.5_Si_14_B_9_ alloy on temperature during heating and cooling [65]. The arrows in the figure indicate the Curie points of crystalline phases, which are precipitated during crystallization [66,67,68,69]. A sharp drop in the magnetic susceptibility during heating at a temperature of 575 K corresponds to the Curie point of the alloy in the amorphous state. The increase in susceptibility at a temperature of 820 K is associated with the appearance of ferromagnetism in the crystals of the α-FeSi solid solution. The maximum magnetic susceptibility occurs at a temperature of 840 K, which corresponds to the maximum volume of the crystalline ferromagnetic phase. The temperature 862 K, at which the relative magnetic susceptibility approaches zero, is close to the Curie point of the Fe_80_Si_20_ solid solution [66]. With an increase in temperature to 970 K, further structural transformations occur and they are associated with the crystallization of the residual amorphous phase and the growth of the crystal grain [70].

At the cooling stage, at a temperature of 910 K, an increase in the magnetic susceptibility is observed, which provides the Fe–Si crystalline phase, see Figure 13. The higher Curie point of this phase in comparison with Fe_80_Si_20_ is due to the reduced silicon content in Fe–Si crystals after recrystallization at temperatures above 900 K [71]. The second rise in the magnetic susceptibility at a temperature of 700 K can be associated with the appearance of ferromagnetism in a phase with a composition close to Fe_23_B_6_.

**Figure 14:**
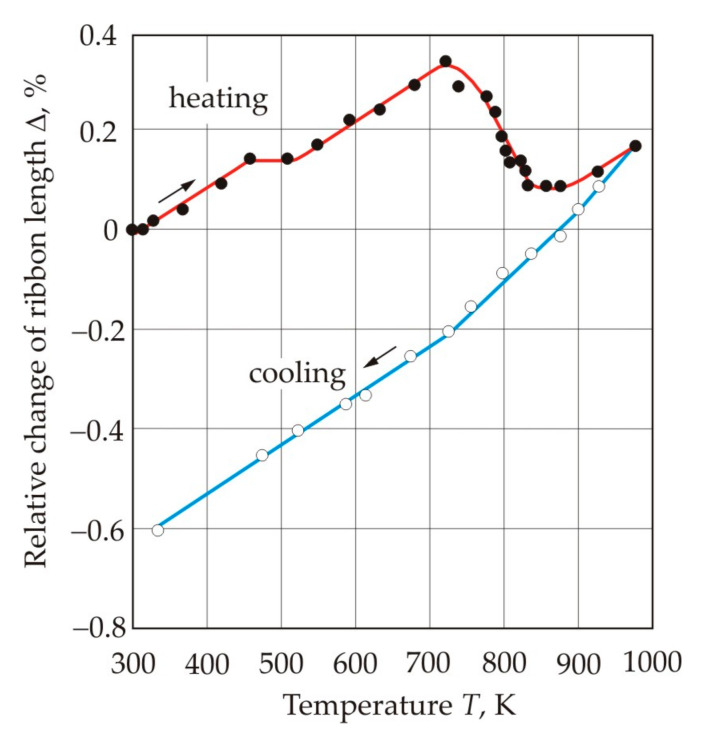
Relative change in the length Δ of a ribbon from the Fe_72.5_Cu_1_Nb_2_Mo_1.5_Si_14_B_9_ alloy as a function of temperature *T* during heating and cooling. Reproduced with permission from [65].

Heat treatment of a nanocrystalline alloy is accompanied by a change in its dimensions associated with structural transformations. Figure 14 shows the relative change in the length Δ of a ribbon made of the Fe_72.5_Cu_1_Nb_2_Mo_1.5_Si_14_B_9_ alloy as a function of temperature *T* during heating and cooling [65]. On the heating curve up to a temperature of 720 K, the length of the ribbon grows almost linearly with the coefficient of linear thermal expansion α*_L_* = 8 × 10^−6^ *K*^−1^. A small area at a temperature of 450–500 K coincides with the onset of structural relaxation of the amorphous precursor [72,73]. An increase in temperature to 820 K leads to a sharp reduction in the length of the ribbon with the coefficient α*_L_* = −30 × 10^−6^ *K*^−1^, which is due to crystallization. The characteristic minimum on the heating curve in the region of 860 K corresponds to the end of the crystallization.

During the cooling, the curve of the length change does not follow the heating curve, even in the high temperature region. This is obvious, since during heating, structural transformations occur continuously, and after they are fixed at the maximum temperature, the structure does not change. Three linear sections can be distinguished on the cooling curve, and the slope of the curves changes at approximately 900 and 700 K. With a decrease in temperature, the coefficient of linear thermal expansion gradually decreases: 18·10^−6^, 13 × 10^−6^ и 10 × 10^−6^ *K*^−1^. The change in the slope of the curves occurs at a temperatures at which the magnetic state of the sample changes in accordance with the cooling curve in Figure 13. Above 900 K, the paramagnetic phase predominates. With a decrease in temperature, the ferromagnetic properties of the Fe-Si crystalline phase appear. At temperatures below 700 K, the crystalline phase of Fe_23_B_6_ becomes ferromagnetic. Thus, the magnetic state of the nanocrystalline alloy has a significant effect on the coefficient of linear thermal expansion of the material.

## 4. Grain Growth Inhibitors

**Figure 15:**
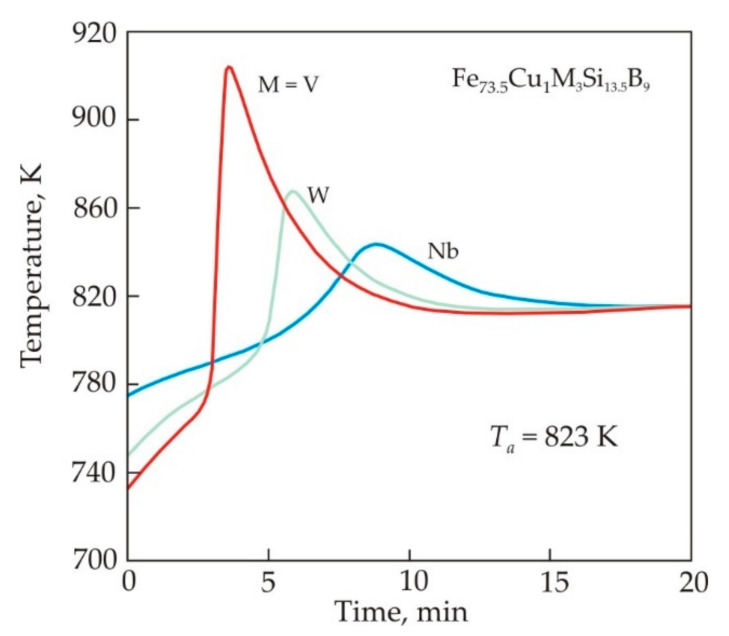
Change in time of the temperature of Fe_73.5_Cu_1_M_3_Si_13.5_B_9_ cores, where M = Nb, W, V, during crystallization. Reproduced with permission from [77].

Grain growth inhibitors can be arranged in the sequence Cr, V, W = Mo, Nb = Ta, Zr, in which each successive element leads to an increase in the crystallization temperature, a decrease in the grain size, and an increase in the permeability of the nanocrystalline alloy [74,75,76]. The crystallization process in alloys containing different inhibitors is very different. Figure 15 shows the change in time of the temperature of Fe_73.5_Cu_1_M_3_Si_13.5_B_9_ cores, where M = Nb, W, V, during crystallization in a furnace with a fixed temperature *T_a_* = 823 K [77]. Crystallization in the alloy with Nb has the lowest peak temperature *T*_max_ = 840 K, the heating rate *V*_max_ = 15 K/min and the highest onset crystallization temperature *T_x_* = 800 K. The values of *T*_max_ and *V*_max_ increase, and *T_x_* decreases in the sequence of inhibitors Nb, W, Mo, V, Cr.

**Figure 16:**
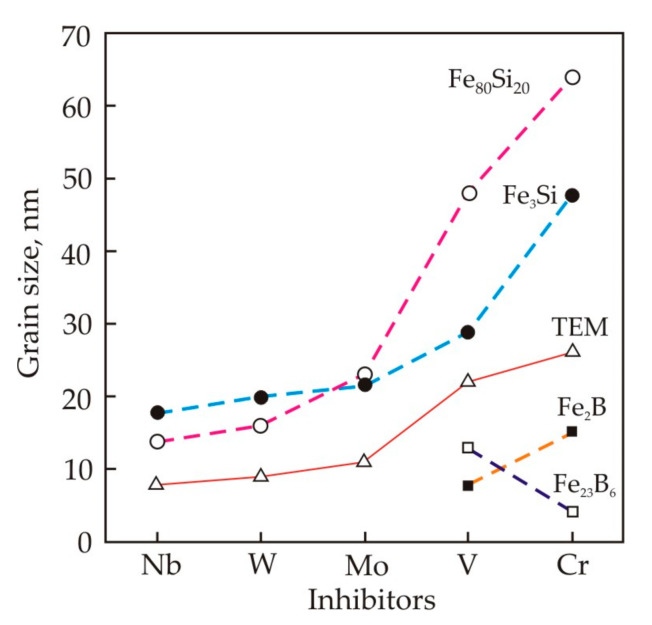
Average grain size from transmission electron microscopy (TEM) and average size of crystalline phases Fe_80_Si_20_, Fe_3_Si, Fe_2_B, and Fe_23_B_6_ from X-ray analysis in Fe_73.5_Cu_1_M_3_Si_13.5_B_9_ alloys with inhibitors M = Nb, W, Mo, V, Cr. Reproduced with permission from [77].

Figure 16 shows that the average grain size measured by a transmission electron microscope (TEM) grows in the sequence of inhibitors in Nb, W, Mo, V, Cr [77]. X-ray diffraction analysis confirms the relationship between the grain size and inhibitors for both the Fe_80_Si_20_ solid solution and the Fe_3_Si phase.

**Figure 17:**
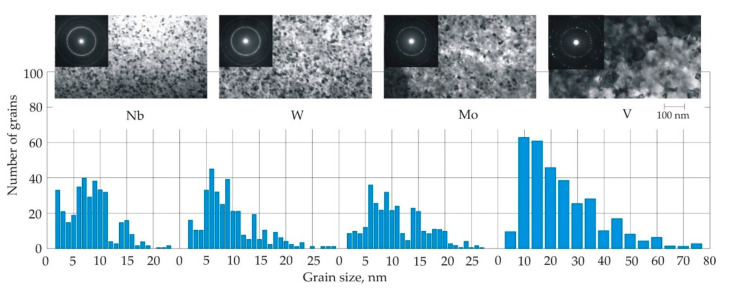
Histograms of grain size distribution and corresponding bright-field images with microdiffraction patterns in Fe_73.5_Cu_1_M_3_Si_13.5_B_9_ alloys with inhibitors Nb, W, Mo, V. Reproduced with permission from [77].

The histograms of grain size distribution in Fe_73.5_Cu_1_M_3_Si_13.5_B_9_ alloys with inhibitors Nb, W, Mo have several peaks, see Figure 17 [77]. In an alloy with Nb, the first peak falls at a size of 2 nm, but this peak is weakly expressed in an alloy with W and is absent in an alloy with Mo. In alloys with Nb, W, Mo, no grains larger than 30 nm were found. In alloys with V and Cr, the average grain size exceeds 20 nm, and the largest grains reach 80 nm. In these alloys, in addition to the Fe_80_Si_20_ solid solution and the Fe_3_Si phase, Fe–B grains are also found, see Figure 16.

**Figure 18:**
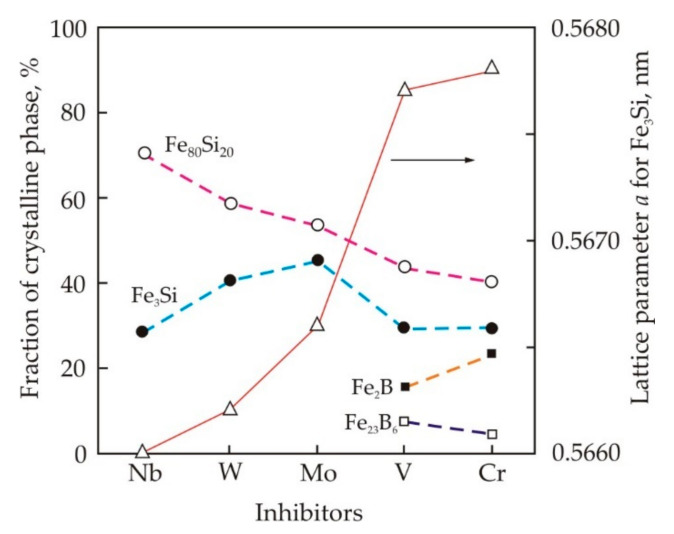
The volume fraction of various crystalline phases and the lattice parameter *a* for Fe_3_Si phase in Fe_73.5_Cu_1_M_3_Si_13.5_B_9_ alloys, where M = Nb, W, Mo, V, Cr. Reproduced with permission from [77].

Figure 18 shows the volume fraction of various crystalline phases in Fe_73.5_Cu_1_M_3_Si_13.5_B_9_ alloys, where M = Nb, W, Mo, V, Cr, while the volume of all crystalline phases is taken as 100% [78]. It follows from the figure that the fraction of the Fe_80_Si_20_ solid solution decreases in the sequence Nb, W, Mo, V, Cr. The fraction of the Fe_3_Si phase increases due Si diffusion in the Fe_80_Si_20_ solid solution. In alloys with V and Cr, the fraction of the Fe_3_Si phase decreases due to the appearance of borides. Figure 18 also shows the dependence of the lattice parameter *a* for Fe_3_Si phase after annealing at 823 K. According to [79], the lattice parameter of the pure Fe_3_Si phase with the DO_3_ structure is 0.5655 nm. In nanocrystalline alloys, the lattice parameter continuously increases in the sequence of Nb, W, Mo, V, Cr, and this indirectly indicates an improvement in the solubility of inhibitors.

The most effective inhibitors have large atomic sizes [80,81]. According to the Hume–Rothery rule [82], the solubility limit occurs when the size of the dissolved atoms is 15% larger than the solvent atoms. In accordance with this criterion, Nb are practically insoluble in αFe at 1000 K, and the solubility of other elements increases in the sequence Nb, W, Mo, V, Cr. With an increase in the solubility, the activation energy of diffusion naturally decreases.

Crystallization is accompanied by an increase in the volume of the crystalline phase due to the movement of grain boundaries. Impurity atoms interact with the moving boundary. The boundary either attracts these atoms, and its speed of motion does not exceed the diffusion rate, or it breaks away from the atoms [83]. At a low migration rate, atoms with a lower diffusion mobility create a stronger inhibition, concentrating near the front of the moving boundary and not dissolving in the crystalline phase. Thus, the effect of inhibition of grain boundaries gradually weakens in the sequence Nb, W, Mo, V, Cr. The weakening of the inhibition is manifested in a decrease in the crystallization temperature *T_x_*, as well as in an increase in the heating rate during crystallization *V*_max_ and the peak temperature *T*_max_. A change in the migration rate of grain boundaries affects the depth of the crystallization process. Thus, the alloy with Nb has a structure that is typical for the initial stage of crystallization, namely, a large number of fine grains with a size of 2 nm, as well as a significant volume of the Fe_80_Si_20_ solid solution.

Table 3 shows the magnetic properties of nanocrystalline Fe_73.5_Cu_1_M_3_Si_13.5_B_9_ alloys with different inhibitors after annealing at *T_a_* = 823 K, 1 h [77]. It follows from the Table that all alloys, with the exception of alloys with V and Cr inhibitors, have high permeability and low coercive force. For the production of nanocrystalline soft magnetic materials, Mo, W and V are also used together with Nb [84,85,86,87,88]. It is known [89] that fine grain and high permeability are obtained after holding the core at a very high temperature, more than 870 K, but for a short time, i.e., the crystallization peak should be high and narrow. The height and width of the crystallization peak also depends on the dimensions of the core. Therefore, the size of the core can also affect the choice of the optimal chemical composition and crystallization regime.

Table 3 also shows that the drop in the initial permeability or disaccommodation 90 min after heat treatment is much lower in an alloy with Nb than in alloys containing W and Mo. The addition of Nb also reduces disaccomodation in alloys with a combined inhibitor system. The drop in permeability in a nanocrystalline alloy can be associated with the redistribution of vacancies in ferromagnetic nanocrystals containing impurity atoms [90] (pp. 537–551), [91]. Atoms of inhibitory elements occupy vacant positions in the substitutional solid solution, and large atoms contribute to a more intense formation of vacancies. Vacancies in nanocrystals can also pass from the amorphous matrix [92]. After demagnetization, which is accompanied by the motion of domain walls, vacancies in nanocrystals tend to occupy new stable states corresponding to new positions of domain walls. Redistributing, vacancies stabilize new positions of domain walls and cause a decrease in permeability. In an alloy with Nb, only a small number of inhibitor atoms are dissolved in α-FeSi and, accordingly, a smaller number of vacancies are formed.

Vacancies are concentrated near inhibitory atoms, promoting the formation of vacancy clusters, which can later grow into voids [39]. This process is most intense at elevated temperatures and can lead to aging. Domain walls are pinned at stable vacancy clusters, promoting an increase in the coercive force and a decrease permeability [90] (pp. 480–491). On the other hand, W atoms at a low concentration of 1–2 at. % decrease the lifetime and stability of vacancy clusters [39]. This feature has a positive effect on the thermal stability of the nanocrystalline alloy with W [93].

**Figure 19:**
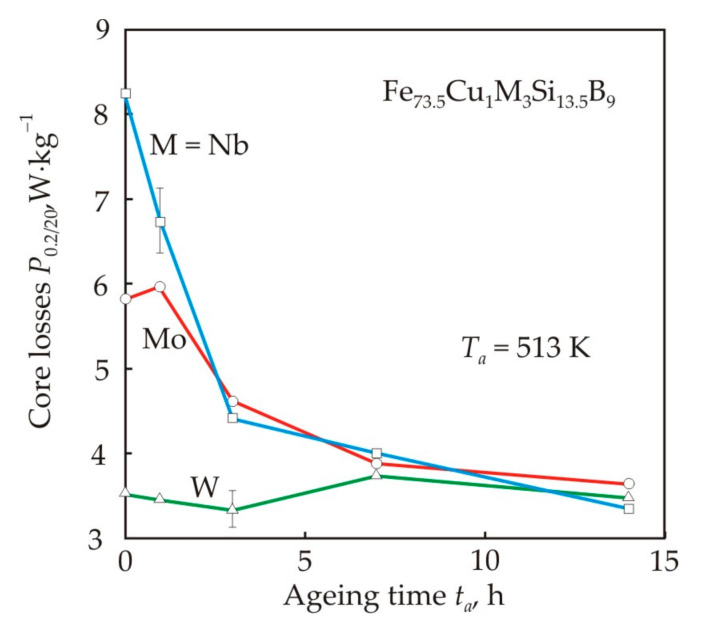
Dependences of the core losses *P*_0.2/20_ (*B_m_* = 0.2 T, *f* = 20 kHz) in Fe_73.5_Cu_1_M_3_Si_13.5_B_9_ alloys, where M = Nb, W, Mo, on the aging time *t_a_* at a temperature of *T_a_* = 513 K. Reproduced with permission from [93].

The vacancy mechanism can be the reason for the decrease in core losses during aging of the nanocrystalline alloy, see Figure 19. In an alternating magnetic field, vacancy clusters can contribute to the domain refinement and a decrease in eddy current losses. At a high frequency, eddy current losses in a metallic magnetic material predominate, for example, their fraction at a frequency of 20 kHz in a nanocrystalline material with a thickness of 20 μm exceeds 80% [94]. A decrease in core losses at the initial stage of annealing was previously found in amorphous magnetic alloys [95,96]. It should be noted that immediately after heat treatment, nanocrystalline alloys containing W have the lowest core losses, see Figure 19 and Table 3.

## 5. Solute Elements in α-Fe

**Figure 20:**
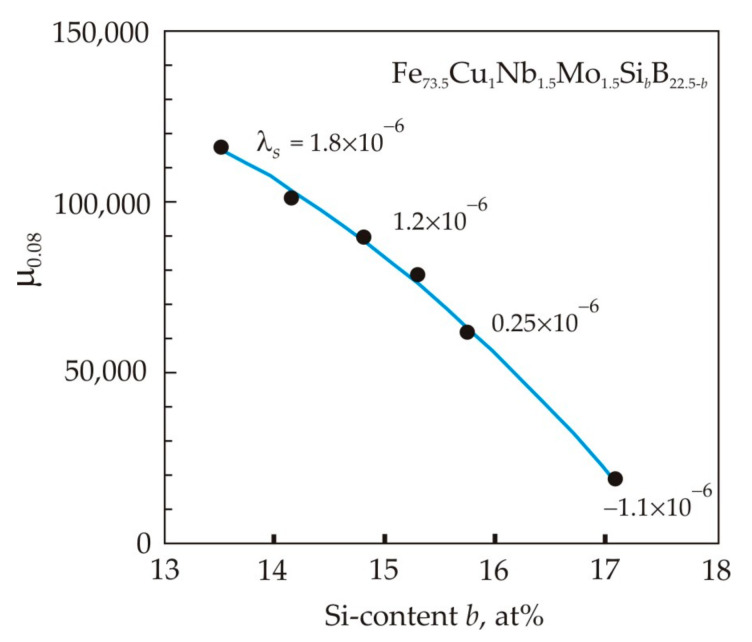
Dependence of the permeability μ_0.08_ on the Si content in the Fe_73.5_Cu_1_Nb_31.5_Mo_1.5_Si_b_B_22.5−b_ alloy. The numbers at the dots indicate the saturation magnetostriction λ_s_ of the corresponding alloys. Adapted from [85] (p. 194).

In the Fe_73.5_Cu_1_Nb_3_Si_13.5_B_9_ alloy, Si is the soluble element in α-Fe. The Si content in nanocrystals plays a significant role in the formation of magnetic properties. In the Fe–Si binary alloy, an increase in the Si content to 11 at. % leads to a decrease in the crystallographic anisotropy constant *K*_1_, and the saturation magnetostriction λ*s* approaches zero [97]. In α-Fe_80_Si_20_ crystals, the magnetostriction takes on a negative value of −6 × 10^−6^, and in the Finemet alloy the residual amorphous phase has a positive magnetostriction of about +20 × 10^−6^. Thus, to obtain a nanocrystalline alloy with a magnetostriction close to zero, a sufficiently large volume of the crystalline phase is required, which would compensate for the positive magnetostriction of the amorphous phase [15]. Taking into account that the volume fraction of the crystalline phase is about 0.7, the silicon content in a nanocrystalline alloy with zero magnetostriction should be about 16 at. %. It follows from Figure 20 that with an increase in the Si content in the Fe_73.5_Cu_1_Nb_31.5_Mo_1.5_Si_b_B_22.5−b_ nanocrystalline alloy, the magnetostriction approaches zero, and the permeability decreases [85] (p. 194).

**Figure 21:**
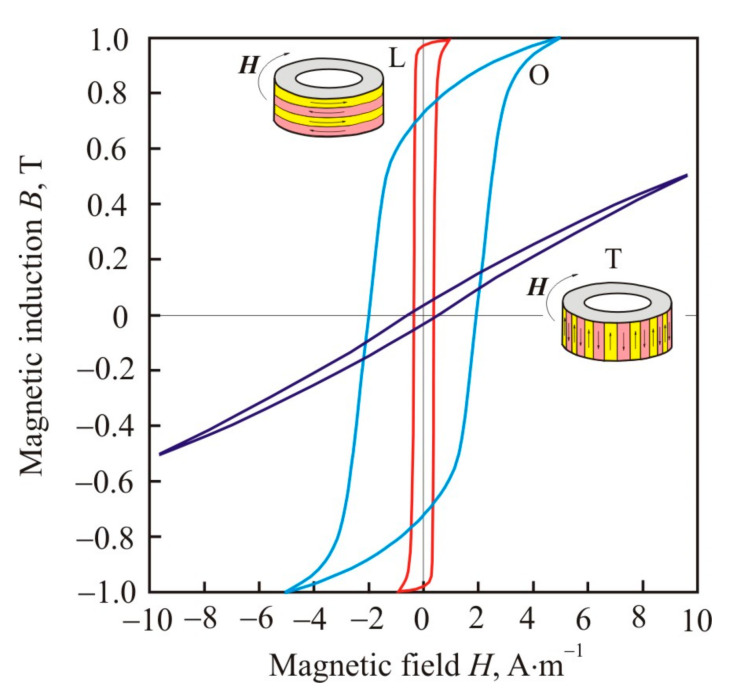
Magnetic hysteresis loops in the cores of the nanocrystalline Fe_67.5_Co_5_Cu_1_Nb_2_Mo_1.5_Si_14_B_9_ alloy after heat treatment in a longitudinal (L) and transverse (T) magnetic field, as well as without a magnetic field (O). A stripe domain structure is shown schematically on the side faces of the L and T cores. Curved arrows show the direction of the external magnetic field during magnetization. Reproduced with permission from [100].

Magnetic-field-induced anisotropy is of a diffusion nature and is associated with directional ordering of Si-atom pairs [90] (pp. 299–309). Diffusion ordering occurs most intensely at high temperatures, but below the Curie point. Typical values of the annealing temperature of 813 K, Curie points of the crystalline phase at 873 K, and of the amorphous matrix at 573 K confirm that Fe–Si grains are the source of magnetic-field-induced anisotropy in the Finemet alloy [98]. During heat treatment, pairs of Si–Si atoms are oriented predominantly along the axis marked by an external magnetic field, and after cooling, this short-range order is retained with the formation of a stripe domain structure with 180° Bloch walls [99]. After annealing in a longitudinal magnetic field, the core has a square hysteresis loop (L) with high remanence ratio, and in a transverse field it has the flat shaped loop (T) with low remanence ratio, see Figure 21 [100]. After annealing without a magnetic field, pairs of Si–Si atoms are oriented in accordance with the local magnetic field, which is randomly oriented. This random ordering leads to local magnetic inhomogeneity and an increase in the coercive force, see loop O in Figure 21. The remanence ratio of round hysteresis loop is around 50%, typical for randomly oriented uniaxial anisotropy.

In the Fe–Si binary alloy, the induced magnetic anisotropy constant *K_u_* takes the highest value at Si = 8–10 at. % [101,102], and the constant decreases with an increase in the Si content. The decrease in *K_u_* with increasing Si content is associated with the formation of a Fe_3_Si superlattice. The phase field of DO_3_-type Fe_1–_*_x_*Si*_x_* alloys extends over the composition range 0.15 < *x* < 0.25 [103]. When *x* = 0.25, all lattice sites for the Fe and Si atoms will be occupied and therefore there is no freedom for an orientational ordering. The magnetic-field-induced anisotropy in Fe–Si nanocrystals of the Finemet alloy fully corresponds to the theory for the Fe–Si alloy, if we take into account the volume fraction of the crystalline phase [15,98]. With an increase in the external magnetic field, the ordering improves and the magnetic anisotropy constant increases [104].

**Figure 22:**
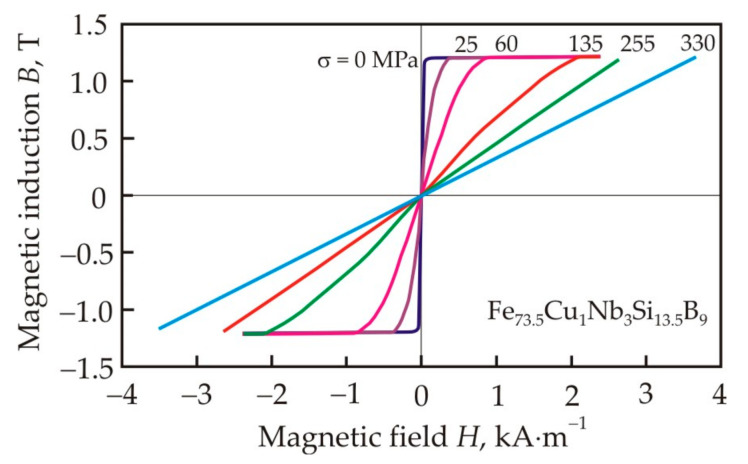
Magnetic hysteresis loops of the nanocrystalline Fe_73.5_Cu_1_Nb_3_Si_13.5_B_9_ alloy after heat treatment under tension. The numbers next to the curves show tensile stress σ during nanocrystallization. Adapted from [105].

Stress annealing of the Fe_73.5_Cu_1_Nb_3_Si_13.5_B_9_ alloy leads to the formation of an in-plane magnetic anisotropy with easy axis perpendicular to the stress axis, which in the ribbon plane can be considered as uniaxial anisotropy with *K_u_* < 0 [105]. The in-plane stress-induced anisotropy confirms the transverse domain structure with zigzag domain walls [106]. Figure 22 shows the hysteresis loops of the Fe_73.5_Cu_1_Nb_3_Si_13.5_B_9_ nanocrystalline alloy after annealing at 803 K for 1h under the action of tensile stresses [105]. It can be seen that, with increasing stress, the slope of flat loops increases and this indicates a decrease in permeability.

It was found in [107] that at a Si content of about 11 at. % in the Fe_87−*x*_Cu_1_Nb_3_Si_x_B_9_ alloy, the type of stress-induced magnetic anisotropy changes. At a high Si content, transverse anisotropy with a flat loop is induced, and at a low Si content, longitudinal anisotropy with a square loop is induced. Nanocrystallization occurs under tension; therefore, after cooling and removal of the load, the crystal lattice of nanograins remains deformed and deformation is due to the internal stress. The interplanar spacing increases along the direction of the tensile stress, and decreases in the transverse direction. Direct evidence for permanent deformation in a nanocrystalline alloy was obtained in [108]. Internal stress will orient the magnetization in a direction that depends on the sign of magnetostriction. Since Fe–Si nanocrystals with a low Si content have positive magnetostriction, then in the Fe_87−*x*_Cu_1_Nb_3_Si_x_B_9_ alloy, longitudinal magnetic anisotropy will form with the direction of magnetization along the tension axis. At a high Si content, the magnetostriction of nanocrystals is negative and transverse magnetic anisotropy with magnetization across the tension axis will form. Stress-induced anisotropy is associated with the magnetostriction of the crystalline phase and internal stresses and does not depend on the presence of free pairs of Si–Si atoms, as in magnetic-field-induced anisotropy.

**Figure 23:**
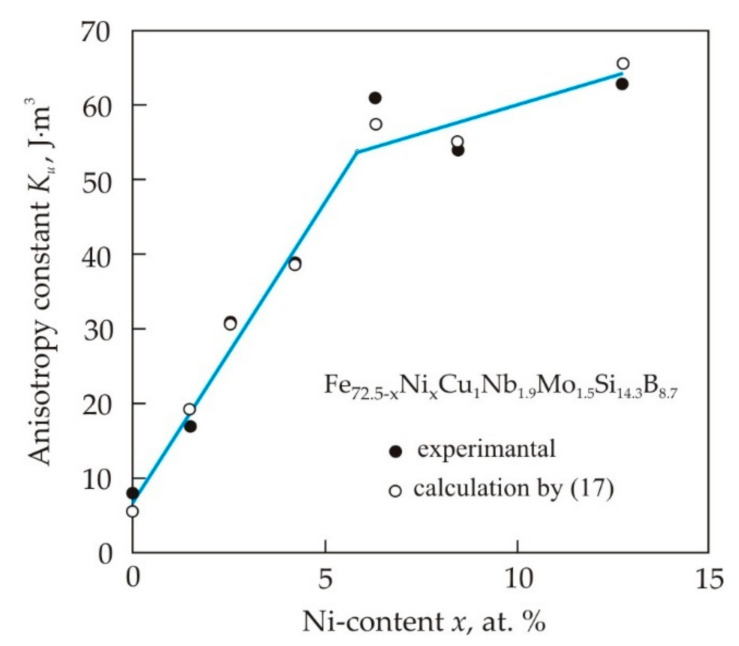
Dependence of the magnetic anisotropy constant *K_u_* on the Ni content in the Fe_72.5−*x*_Ni*_x_*Cu_1.1_Nb_1.9_Mo_1.5_Si_14.3_B_8.7_ alloy calculated from the magnetization work and from the initial permeability μ*_i_* by formula (17). Reproduced with permission from [110].

The introduction of Ni and Co soluble in α-Fe into a nanocrystalline alloy leads to an increase in the magnetic anisotropy constant *K_u_* induced in a magnetic field [109]. Figure 23 shows the dependence of the magnetic anisotropy constant *K_u_* on the Ni content in the Fe_72.5−*x*_Ni*_x_*Cu_1.1_Nb_1.9_Mo_1.5_Si_14.3_B_8.7_ alloy [110]. Linearity of the curve up to a Ni content of 7 at. % indicates that all newly dissolved Ni atoms contribute to the uniaxial induced anisotropy, participating in pair ordering. The weaker growth of *K_u_* can be associated with a decrease in free pairs of atoms capable of rearranging in a magnetic field. It also follows from Figure 23 that the experimental constant determined from the magnetization work agrees well with the constant determined from the initial permeability μ*_i_* in the model of magnetization rotation:

(17) $$K_{u}=\frac{B_{s}^{2}}{2\mu_{0}\mu_{i}}.$$

**Figure 24:**
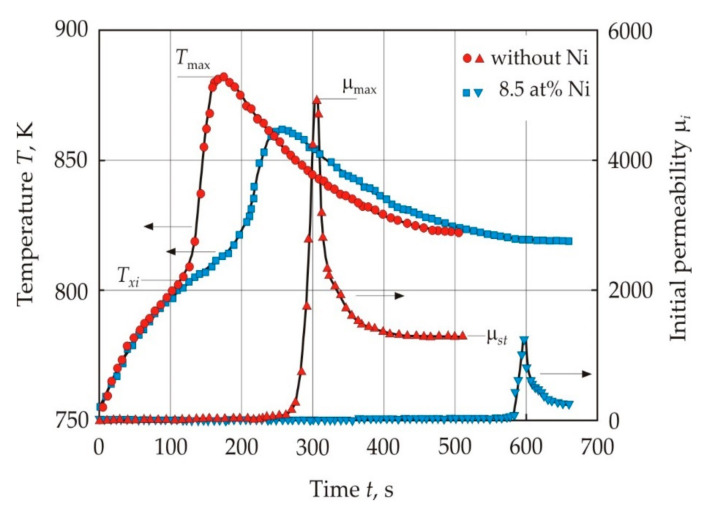
Time dependences of core temperature *T* and initial permeability μ*_i_* in the crystallization process of Fe_72.5−*x*_Ni*_x_*Cu_1_Nb_2_Mo_1.5_Si_14_B_9_ alloys without Ni and with a Ni content of 8.5 at% at furnace temperature 823 K. Reproduced with permission from [111].

Figure 24 shows the dependences of temperature *T* and initial permeability μ*_i_* at a frequency of 1 kHz on time *t* during crystallization of Fe_72.5−*x*_Ni*_x_*Cu_1_Nb_2_Mo_1.5_Si_14_B_9_ alloys with *x* = 0 and 8.5 at. % in a furnace with a fixed temperature of 823 K [111]. Nickel atoms in α-Fe have a low diffusion mobility and their solubility at 700 K does not exceed 5 at. % [112]. The smoothing of the crystallization peak in an alloy with Ni is similar to the effect of inhibitors, see Figure 15. The low diffusion mobility and limited solubility of Ni suggest that insoluble Ni atoms also have an inhibitory effect on crystal growth. The increased Ni content inhibits the formation of the crystalline phase, and the volume fraction of the amorphous phase increases [111]. X-ray analysis shows [111,113] that Ni in the crystalline phase is concentrated mainly in the Fe_3_Si superlattice, partially replacing Fe atoms. Prolonged annealing of the alloy with Ni is accompanied by a strong increase in the coercive force [114], which can be associated with the formation of the tetragonal phase Fe_3_NiSi_1.5_ during the slow diffusion of Ni atoms [113].

Figure 24 also shows the time dependence of permeability, which was measured continuously together with the core temperature [111]. As the Ni content increases, the temperature for the peak permeability μ_max_ decreases. This indicates an increase in the Ni content in the crystalline phase, since Ni lowers the Curie point of Fe-based alloys.

Another soluble element in α-Fe is Al, which is used in non-oriented electrical steel and in the sendust alloy Fe_73_Si_17_Al_10_ with high permeability and low crystallographic magnetic anisotropy constant [115]. Partial substitution of Fe with Al improves magnetic properties of the nanocrystalline Fe_73.5−x_Al_x_Cu_1_Nb_3_Si_13.5_B_9_ alloy [116].

Silicon plays an important role in the creation of a high permeability nanocrystalline alloy. Despite the rather high saturation magnetization *J_s_* = 1.25 T, the Fe_73.5_Cu_1_Nb_3_Si_13.5_B_9_ alloy cannot compete with the grain-oriented electrical steel, which has *J_s_* = 2.03 T and is used at an industrial frequency of 50–60 Hz in power transformers. The low saturation magnetization in the Finemet is associated with the high Si content in the nanocrystals. To increase *J_s_*, it is necessary to exclude as much as possible soluble nonmagnetic atoms in the crystal grain. In addition, a nanocrystalline material for industrial frequency should have a coercive force *H_c_* of no more than 6 A·m^−1^, this value corresponds to a typical coercive force for a grain-oriented electrical steel. Nanocrystalline soft magnetic materials have their advantages over electrical steel. Approximately two times higher electrical resistance and an order of magnitude less thickness give a significant reduction in eddy current losses.

At the stage of the preceding crystallization of a nanocrystalline alloy with an increased Fe content, the homogeneous amorphous matrix is separated into regions with different chemical compositions [117]. Crystallization of such regions leads to the formation of crystallites, which differ in size and magnetic anisotropy constants. In an alloy with a low Si content, the crystallization temperature decreases and the grain size increases, and this is associated with a weakening of the growth inhibition of Si-depleted grains. In nanocrystalline alloys with high saturation magnetization, Si is not used or is used as a small additive. To inhibit grain growth, Zr and B [118], P and B [119,120] or B alone [121,122] are used. To obtain finer grain an increase of heating rate is used during annealing [89,122,123]. Rapid heating at elevated temperatures allows the number of Cu-clusters to almost double [124], and a grain is formed whose size is about 30% smaller than with slow heating. In α-Fe nanocrystals in the absence of silicon, it is also possible to obtain a large magnetic anisotropy constant after annealing in a magnetic field, and this is due to the anisotropic distribution of interstitial atoms B, Zr, and Nb [125].

**Figure 25:**
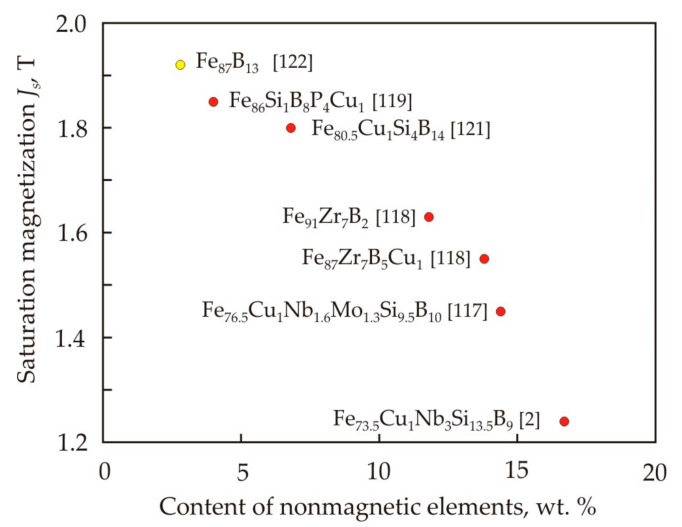
Dependence of saturation magnetization *J_s_* on the mass content of non-magnetic elements in nanocrystalline soft magnetic alloys with a coercive force less than 6 A⋅m^−1^. The Fe_87_B_13_ alloy was obtained at a heating rate of 3 K·s^−1^.

Figure 25 shows the dependence of the saturation magnetization *J_s_* on the mass content of non-magnetic elements in nanocrystalline soft magnetic alloys with a coercive force less than 6 A·m^−1^. The Fe_87_B_13_ alloy was obtained at a high heating rate of 3 K·s^−1^ [123]. It follows from the Figure that the saturation magnetization of the nanocrystalline alloy decreases in proportion to the mass fraction of non-magnetic elements [122].

## 6. Core Losses

Core losses determine the electromagnetic energy absorbed by a magnetic material. The work done by an electromagnetic field over a unit volume of material when the magnetic induction changes from *B*_1_ to *B*_2_ is equal to:

(18) $$A=\int_{B_{1}}^{B_{2}}HdB=HB\left|{}_{B_{1}}^{B_{2}}\right.-\int_{H_{1}}^{H_{2}}BdH,$$

and when changing along a full cycle,

(19) A=−∮BdH=∮HdB.

The quantity of *A* is equal to the area of the magnetic hysteresis loop. A static hysteresis loop corresponds to an infinitely slow magnetization, and a dynamic loop is formed during a magnetization with a frequency *f*. The product of the area of the static hysteresis loop *W_h_*, J·m^−3^, by the frequency *f* is the hysteresis losses *P_h_*, and the product of the area of the dynamic hysteresis loop by the frequency is the total losses *P*.

In essence, the magnetization is the rotation of the atomic magnetic moments, which is accompanied by the precession of magnetization and is described by the Landau–Lifshitz equation [90] (pp. 562–566). Friction leads to damping of precession and the approach of the magnetization vector to the direction of the external magnetic field. Magnetic relaxation is associated with the interaction of carriers of magnetic moments with each other (spin-spin relaxation) and their environment (spin-lattice relaxation). As a result of magnetic relaxation, the energy of motion of the magnetic moments transforms into heat, and the system tends to a state of thermodynamic equilibrium. Relaxation losses are the main source of high-frequency losses in non-metallic magnetic materials; however, in metallic materials against the background of eddy currents, they are in many case insignificant.

The nature of eddy current losses is evident from classical electrodynamics. However, in ferromagnetic materials, it is necessary to take into account the features caused by the magnetic domain structure. In a ferromagnet, a change in magnetic induction occurs only near a moving domain wall, and eddy currents are also concentrated here. Therefore, with an increase in the size of the domains, the inhomogeneity of the magnetization increases. The eddy current losses *P_ed_*, W·kg^−1^, in the model of flat domain walls are proportional to the domain width *D* [126] and under the condition *D* > *h*, *h* is the plate thickness, m, they are equal to:

(20) $$P_{ed}\approx 1.628\frac{D}{h}P_{c},$$

where *P_c_* is the classic eddy current losses for a uniform magnetic flux

(21) $$P_{c}=\frac{\pi^{2}B_{m}^{2}h^{2}f^{2}}{6\rho }$$

and *B_m_* is the amplitude of sinusoidal magnetic induction, T, *f* is the frequency, Hz, ρ is the electrical resistivity, Ω·m. If the domain width is less than the ribbon thickness, then the magnetic flux in the material is more uniform, and *P_ed_* approach the classical eddy current losses *P_c_*.

**Figure 26:**
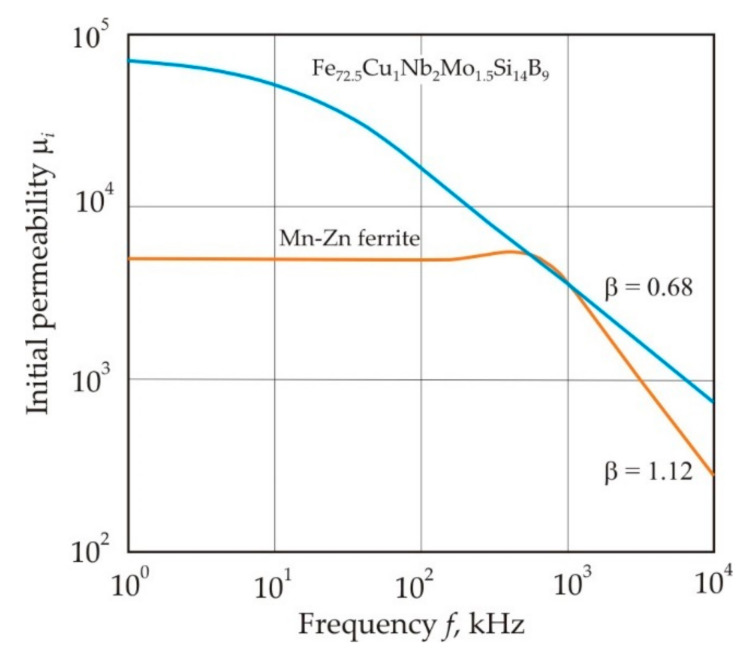
Frequency dependence of the initial permeability μ*_i_* in nanocrystalline Fe_72.5_Cu_1_Nb_2_Mo_1.5_Si_14_B_9_ alloy and Mn–Zn ferrite. Reproduced with permission from [19] (p. 69).

The influence of eddy currents and magnetization relaxation illustrates the frequency dependence of the permeability in a metal nanocrystalline alloy and ferrite, see Figure 26 [19] (p. 69).

In the high-frequency region, the exponent β of the power function:

(22) $$\mu_{i}\propto f^{-\beta }$$

equal to 0.68 and 1.12 for the metal Fe_72.5_Cu_1_Nb_2_Mo_1.5_Si_14_B_9_ alloy and Mn-Zn ferrite, respectively, i.e., in ferrite, the decrease in μ*_i_* with frequency is steeper. These exponents are close to 0.5 for the penetration depth of the magnetic field:

(23) $$\delta_{H}=\sqrt{\frac{\rho }{\pi \mu \mu_{0}f}}$$

which decreases under the influence of eddy currents, and 1.0 for the frequency dependence of the permeability caused by the magnetization relaxation,

(24) $$\mu =\mu_{i}\frac{\omega_{0}^{2}}{2\lambda \omega }$$

where λ and ω_0_ are the damping coefficient and natural frequency of the domain wall oscillation [85] (pp. 123–127).

Hysteresis is associated with the existence of stable and metastable states in the material and with irreversible transitions between these states. In soft magnetic materials, irreversible displacement of domain walls is the main cause of hysteresis. During magnetization, the domain wall or its individual sections jump into new equilibrium states. Such a jump is accompanied by a local change in the magnetic induction and the corresponding local eddy currents. The time interval between jumps depends on the density of defects and the velocity of motion of domain walls. If the duration of a jump is less than the time interval between two successive jumps, then the overlap of eddy currents from adjacent jumps does not occur. Domain wall jumps can occur simultaneously and not overlap due to their remoteness in space. In this case, the magnetic losses per cycle are equal to the sum of the eddy current losses arising from individual jumps. These local eddy current losses are frequency independent and are essentially hysteresis losses.

Eddy currents caused by jumps begin to overlap in time and space with an increase in the velocity of domain wall movement, i.e., with increasing frequency and induction, and this leads to the dependence of magnetic losses on frequency.

**Figure 27:**
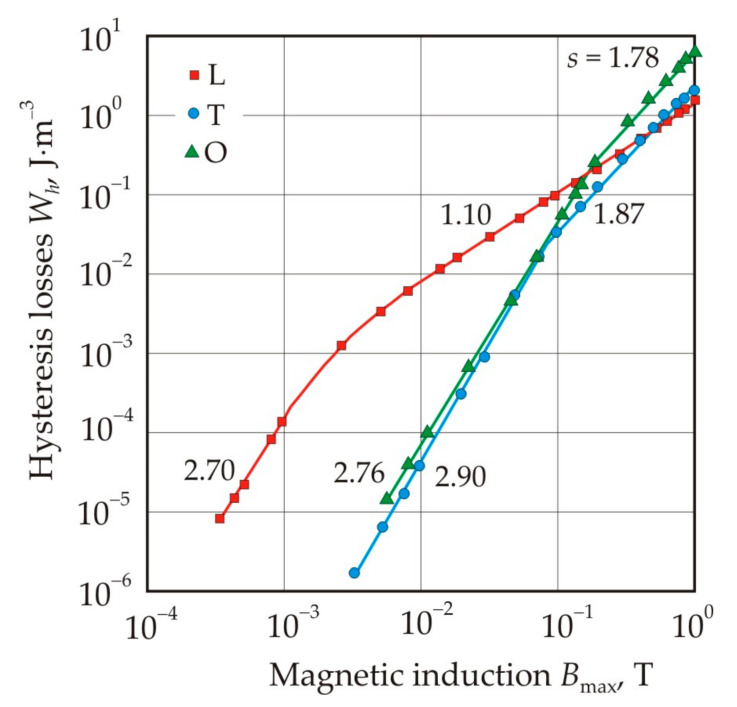
Dependences of hysteresis losses *W_h_* on the maximum induction *B*_max_ after heat treatment in a longitudinal (L) and transverse (T) magnetic field, as well as without a magnetic field (O), for cores made of nanocrystalline Fe_67.5_Co_5_Cu_1_Nb_2_Mo_1.5_Si_14_B_9_ alloy. The numbers on the curves represent the exponent *s* for the power function (25). Reproduced with permission from [100].

Figure 27 shows the dependences of the hysteresis losses *W_h_* on the maximum induction *B*_max_ after heat treatment in a longitudinal (L) and transverse (T) magnetic field, as well as without a magnetic field (O), for cores made of a nanocrystalline Fe_67.5_Co_5_Cu_1_Nb_2_Mo_1.5_Si_14_B_9_ alloy [100]. On the curves in the logarithmic scale, one can select linear sections that correspond to a constant exponent *s* for the approximating power function:

(25) $$W_{h}=rB_{\max}^{s}$$

The power function is scale invariant. This means that all power functions with the same exponent s are equivalent to each other and differ only in scale, i.e., in the coefficient *r*. Thus, the constant *s* in a certain interval of the independent variable, in our case *B*_max_, can be associated with the invariability of the process described by this power function. Conversely, a change in the exponent *s* should indicate a qualitative change in this process.

**Figure 28:**
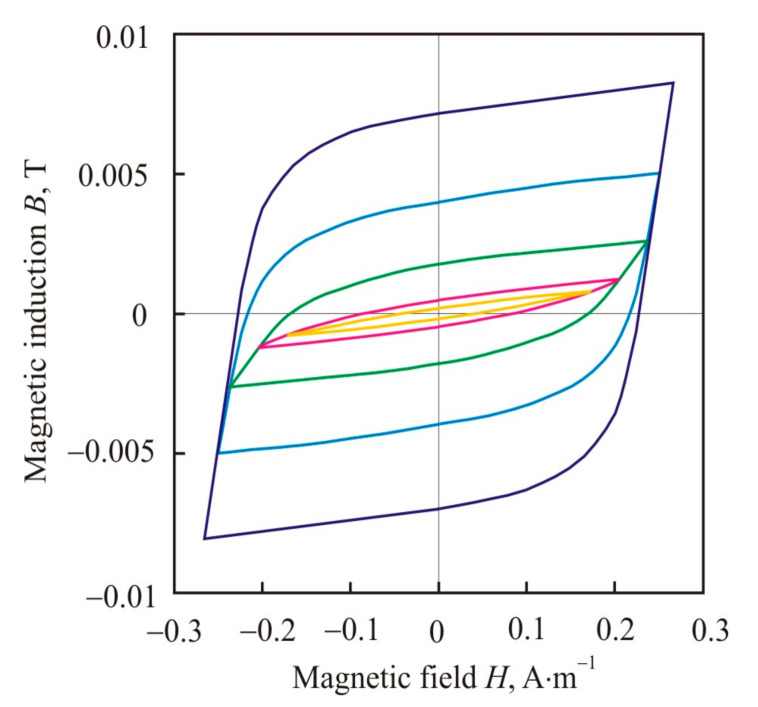
Minor magnetic hysteresis loops in the L core with longitudinal induced anisotropy for the nanocrystalline Fe_67.5_Co_5_Cu_1_Nb_2_Mo_1.5_Si_14_B_9_ alloy in a weak magnetic field. Reproduced with permission from [100].

As can be seen from Figure 27, in a weak magnetic field, the core L with longitudinal uniaxial anisotropy has the greatest hysteresis losses [100]. In this core, at *B*_max_ = 0.0025 T, the exponent *s* changes the value 2.70, which is close to exponent 3 in Rayleigh’s law, by 1.10. At the same magnetic induction, the hysteresis loop changes the lens-like shape with a low remanence ratio, which is typical for the Rayleigh region, to a square one, see Figure 28.

The change in the shape of the hysteresis loop can be interpreted as follows. The loop has a square shape if most of the domain walls almost simultaneously overcome the maximum local potential barriers. In the Rayleigh region, a reversible displacement of domain walls predominates, and this displacement should be small and not exceed the characteristic length of the local magnetic inhomogeneity of the material. In a nanocrystalline alloy, the scale of the magnetic inhomogeneity is related to the correlation length *L_ex_*, i.e., to the size of ferromagnetic clusters. The increment in magnetic induction Δ*B* with displacement of flat domain walls by a distance *L_ex_* is:

(26) $$\Delta B=\frac{2J_{s}}{D}L_{ex}$$

where *D* is the domain width, *J_s_* is the saturation magnetization. For the assessment we use the following values *J_s_* = 1.2 T and *D* = 10^−3^ m [127], correlation length *L_ex_* = 10^−6^ m. These values give an increment in magnetic induction Δ*B* = 0.0024 T, which is close to 0.0025 T obtained from Figure 27 for the Rayleigh region in core L.

If there is a periodic change in the local magnetic energy density Δ*E* in a ferromagnetic material, then the coercive force associated with a delay in the displacement of domain walls at these inhomogeneities can be expressed in the form [90] (pp. 487–491):

(27) $$H_{c}\approx \frac{\Delta E}{\mu_{0}M_{s}}\frac{\delta }{l}$$

where δ is the width of the domain wall, *l* is the characteristic length of the magnetic inhomogeneity. In a nanocrystalline alloy, Δ*E* = $\langle K\rangle$ and *l* ≈ *L_ex_*. The largest coercive force is obtained under the condition *l* = δ. For a nanocrystalline alloy, δ ≈ *L_ex_* and the coercive force associated with the random distribution of the easy axes in ferromagnetic clusters is:

(28) $$H_{c}\approx \frac{\langle K\rangle }{\mu_{0}M_{s}}$$

After annealing in a magnetic field, the easy magnetization axes in ferromagnetic clusters are concentrated in a certain solid angle, which narrows with an increase in the induced magnetic anisotropy constant *K_u_*. In this case, the differences between the orientations of the easy magnetization axes of neighboring clusters are smoothed out. Such smoothing is equivalent to a decrease in the inhomogeneity Δ*E*, and should lead to a decrease in the coercive force. This mechanism explains the decrease in the coercive force in the L core after annealing in a longitudinal magnetic field compared to the O core, which was annealed without a magnetic field.

For core T, the dependence of hysteresis losses on magnetic induction in a weak magnetic field has an exponent *s* = 2.90, which corresponds to Rayleigh’s law, see Figure 27. The exponent does not change up to a magnetic induction of 0.08 T. Thus, the Rayleigh region in the core T is an order of magnitude larger compared with the core L. The value *B*_max_ = 0.08 T separates the regions of uniform reversible rotation of magnetization, typical for a weak magnetic field, and inhomogeneous irreversible rotation.

**Figure 29:**
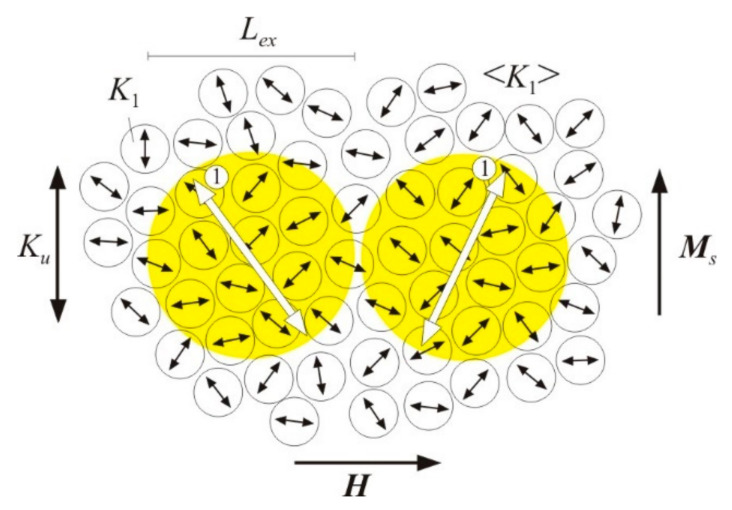
Schematic representation of the irreversible magnetization rotation in a nanocrystalline material with induced transverse magnetic anisotropy. Reproduced with permission from [100].

The mechanism of irreversible magnetization rotation can be as follows [100]. In a nanocrystalline material, the easy magnetization axes of ferromagnetic clusters are randomly distributed. In a material with induced magnetic anisotropy *K_u_*, the easy magnetization axes of clusters are concentrated near the direction of the magnetic field during heat treatment. In Figure 29, double arrows show the axes of easy magnetization of the clusters [100]. The ***M****_s_* vector denotes the direction of magnetization in the magnetic domain. Without an external magnetic field, the magnetization in neighboring clusters is directed towards the number 1 in Figure 29. In an external magnetic field ***H*** directed at an angle of 90° to the easy magnetization axis *K_u_*, the magnetization of the clusters rotates to the direction ***H***. In the left cluster, the rotation from direction 1 will lead to an increase in the angle between the cluster magnetization and its easy magnetization axis, i.e., to an increase in the magnetic anisotropy energy. Therefore, at some moment, direction 1 will become unstable and an irreversible magnetization rotation by 180° in the direction closest to the direction of the external magnetic field will occur. In the right cluster in Figure 29, the magnetization will smoothly rotate towards the direction of the magnetic field. Thus, many ferromagnetic clusters can experience an irreversible magnetization rotation towards the direction of the external magnetic field. The mechanism of irreversible magnetization rotation in a nanocrystalline material is similar to the irreversible rotation that occurs in a particle with uniaxial magnetic anisotropy [90] (pp. 491–498).

Comparison of hysteresis losses for O and T cores in the Figure 27 shows that they practically do not differ up to *B*_max_ = 0.08 T. Further, the curves diverge due to a faster growth of hysteresis losses in the core O. This proves that the mechanisms of magnetic hysteresis in O and T cores in a weak magnetic field are the same or close, and the main role is assigned to reversible rotation of magnetization [128].

From the Rayleigh equations, one can find relations connecting the parameters of the hysteresis loop [129,130], in particular, the hysteresis losses can be calculated by the formulas:

(29) $$W_{h}=\frac{8}{3}B_{\max}H_{c}$$

(30) $$W_{h}=\frac{8}{3}B_{r}H_{\max}$$

(31) $$W_{h}=\frac{8}{3}\sqrt{B_{r}B_{\max}H_{c}H_{\max}}$$

In core O with a round hysteresis loop, the difference between the measured and calculated hysteresis losses for formulas (29)–(31) does not exceed 2% [100]. In L and T cores with induced magnetic anisotropy, the convergence is worse and it is in the range of 8–20% for cores with different types of anisotropy and for different formulas.

**Figure 30:**
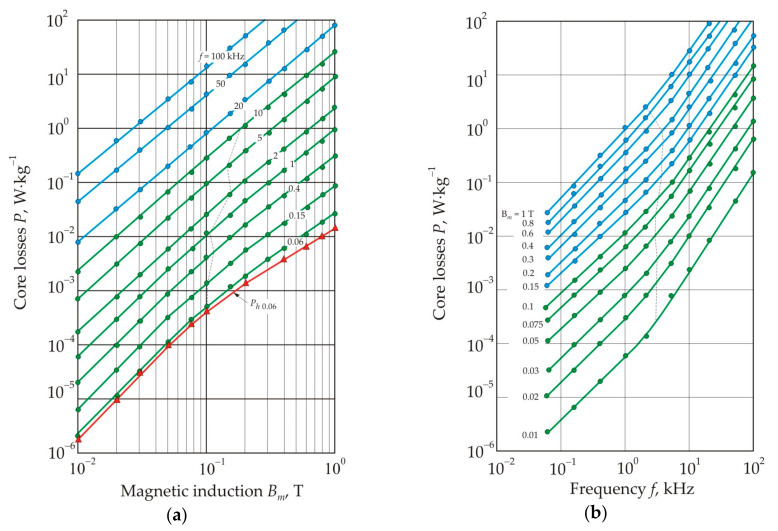
Dependences of core losses *P* on magnetic induction *Bm* at different frequency *f* (**a**) and dependences of core losses *P* on frequency *f* for different magnetic induction *Bm* in the Fe72.5Cu1Nb2Mo1.5Si14B9 alloy after heat treatment without a magnetic field. The numbers on the curves indicate the frequency *f* in kHz (**a**) and the magnetic induction *Bm* in T (**b**). Curve *Ph*60 corresponds to hysteresis losses at a frequency of 60 Hz. The dashed line marks the transition points that correspond to the change in the slope of the linear sections of the curves. Reproduced with permission from [94].

Figure 30 shows the dependences of the core losses *P* on the amplitude of the magnetic induction *B_m_* and the frequency *f* for the nanocrystalline Fe_72.5_Cu_1_Nb_2_Mo_1.5_Si_1_ alloy [94]. It follows from the Figure that the *P*(*B_m_*) dependences on a logarithmic scale have two linear sections with different slopes, and at a frequency above 20 kHz, the curves degenerate into one straight line. The dashed line marks transition points that are close to *B_m_* = 0.1 T. Each curve in the frequency dependence of core losses in Figure 30 also has two linear sections with transition points at a frequency of 3 kHz, at which the skin effect begins to appear in a 25 μm thick ribbon [94].

**Figure 31:**
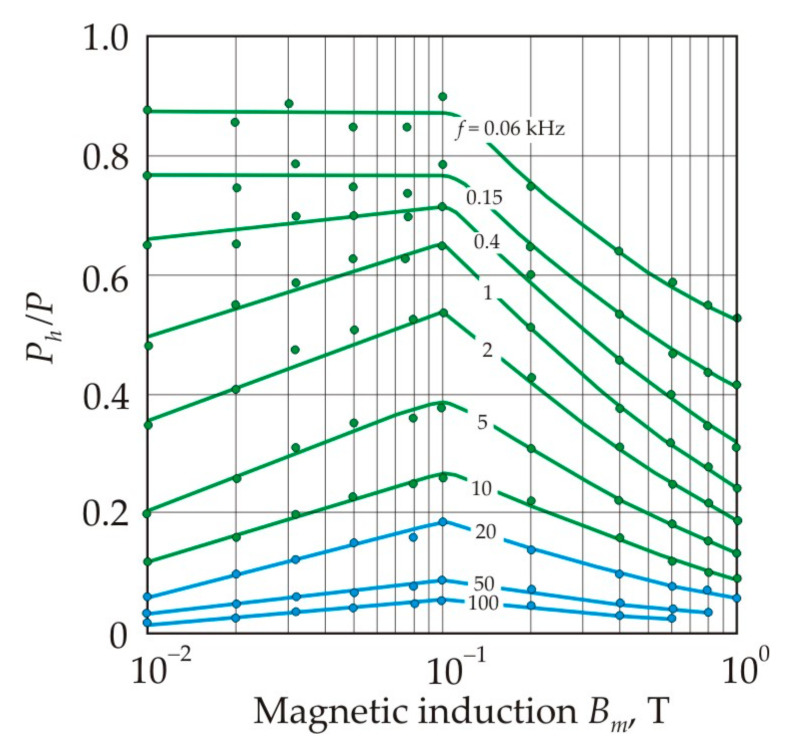
Ratio of hysteresis losses *P_h_* to total magnetic losses *P* as a function of magnetic induction *B_m_* in nanocrystalline alloy Fe_72.5_Cu_1_Nb_2_Mo_1.5_Si_14_B_9_ at different frequency *f*. Reproduced with permission from [94].

Figure 31 shows the dependences of the ratio of hysteresis losses to total magnetic losses *P_h_*/*P* on magnetic induction *B_m_* at different frequencies [94]. Hysteresis losses *P_h_*, W·kg^−1^, were determined from the ratio:

(32) $$P_{h}=\frac{W_{h}}{\rho }f,$$

where ρ is the density. It follows from Figure 31 that hysteresis losses prevail in a weak magnetic field and at a low frequency, and here their fraction reaches 90%. In this region, the total loss curves are close to the hysteresis loss curves, see Figure 30a. Fraction of eddy current losses increases at high magnetic induction *B_m_*, when the motion of domain walls becomes smooth, and in the high-frequency region.

**Figure 32:**
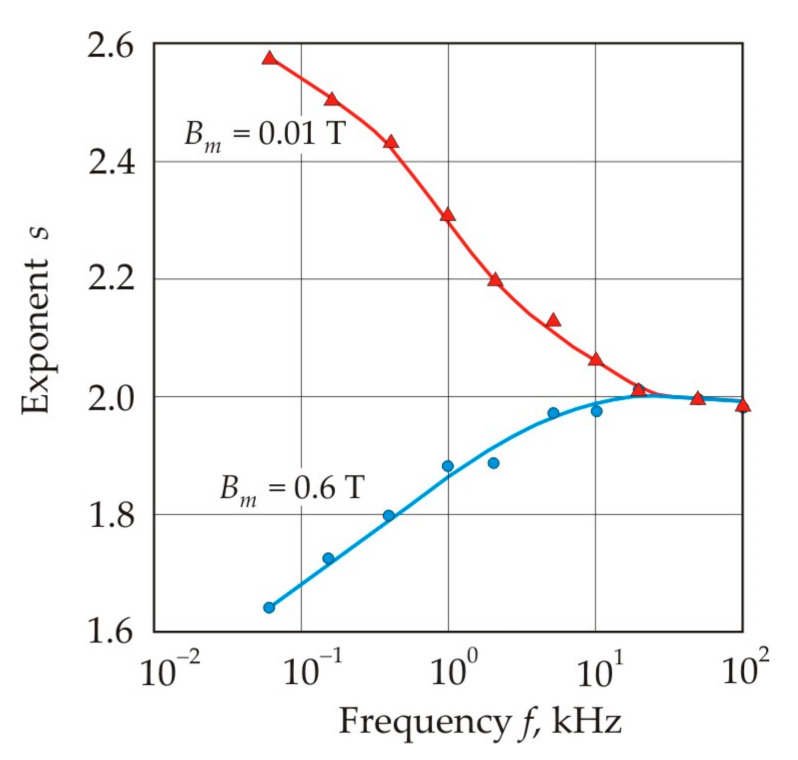
Frequency dependences of the exponent *s* for the approximating power function (25) in the nanocrystalline Fe_72.5_Cu_1_Nb_2_Mo_1.5_Si_14_B_9_ alloy at magnetic induction *B_m_* = 0.01 and 0.6 T. Reproduced with permission from [94].

Figure 32 shows the frequency dependences of the exponent *s* for the approximating power function (25) in a weak (*B_m_* = 0.01 T) and middle (*B_m_* = 0.6 T) magnetic field. In a weak magnetic field, hysteresis losses predominate and the exponent is close to 3, which is characteristic of Rayleigh hysteresis loops. At high frequency, core losses are associated with eddy currents, and the exponent *s* approaches 2 in accordance with formulas (20) and (21). The exponent *s* = 1.6 corresponds to the approximate balance of hysteresis and eddy current losses [131].

**Figure 33:**
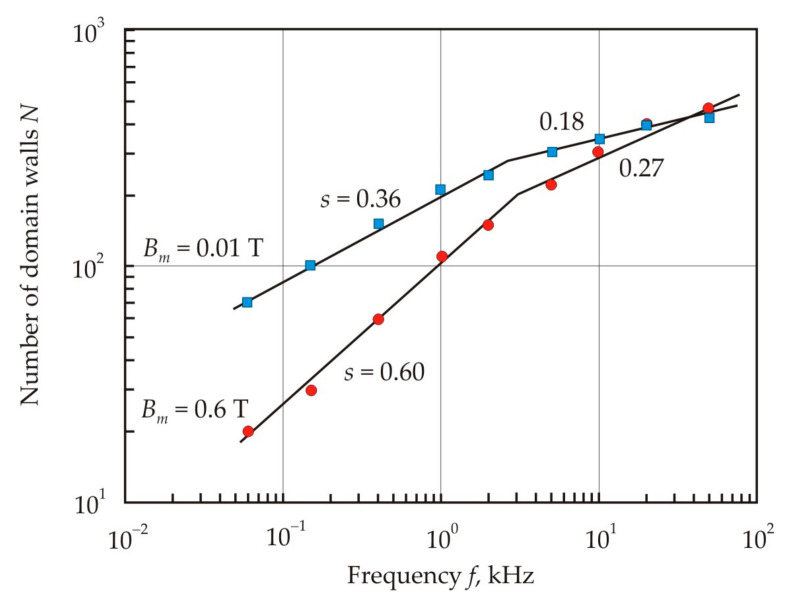
Frequency dependence of the calculated number of domain walls *N* for *B_m_* = 0.01 and 0.6 T in a 10 mm wide ribbon made of nanocrystalline Fe_72.5_Cu_1_Nb_2_Mo_1.5_Si_14_B_9_ alloy. The numbers on the curves show the exponent *s* for the approximating power function. Adapted from [85] (pp. 212–214).

By comparing the measured eddy current losses *P_ed_* = *P* − *P_h_* with formula (20), one can calculate the number of domain walls involved in the magnetization. Calculations show that the number of domain walls increases with increasing frequency, see Figure 33 [85] (pp. 212–214). This fact follows from the principle of minimum entropy production, according to which the frequency dependence of the domain width *D* is a power function of frequency with exponent *s* = −0.5 [132] and accordingly, the exponent *s* = 0.5 for the number of domain walls.

It follows from Figure 33 that the number of domain walls is larger in a weak magnetic field at *B_m_* = 0.01 T. An increase in the number of domain walls can be associated with the special role of ferromagnetic clusters in the absence of induced magnetic anisotropy [133]. The cluster structure of a nanocrystalline material is a magnetic inhomogeneity that promotes the domain refinement in an alternating magnetic field [134,135]. In the low-frequency region at *B_m_* = 0.6 T, there is a smooth motion of domain walls, and the exponent *s* is closest to 0.5. At frequencies above 3 kHz, the skin effect begins to prevail, and the number of domain walls weakly depends on the frequency.

## 7. Applications

**Figure 34:**
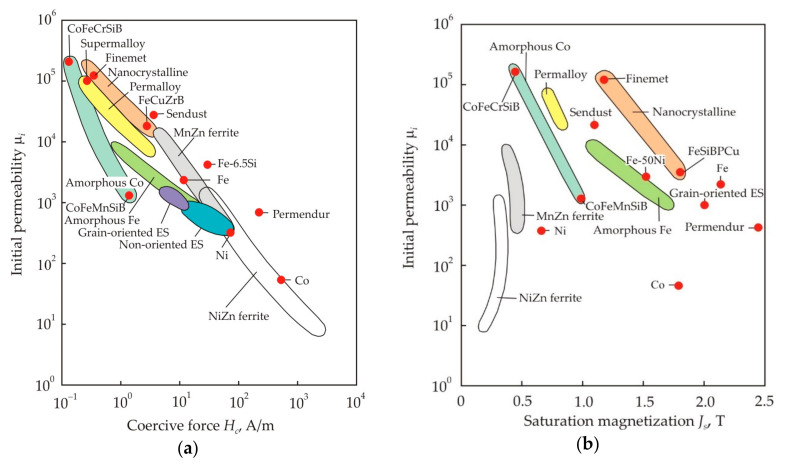
Relationship of initial permeability μ*_i_* with coercive force *H_c_* (**a**) and saturation magnetization *J_s_* (**b**) for soft magnetic materials. Reproduced with permission from [19] (pp. 117–121).

Figure 34 shows the relationship of the initial permeability μ*_i_* with the coercive force *H_c_* (a) and the saturation magnetization *J_s_* (b) for soft magnetic materials [19] (pp. 117–121). The closed curves mark the areas corresponding to various materials of the same name, and the dots mark the most famous soft magnetic materials. It can be seen that a good correlation between μ*_i_* and *H_c_* takes place for a wide variety of soft magnetic materials, ferromagnetic and ferrimagnetic, crystalline, nanocrystalline, and amorphous.

Magnetic materials are used mainly in the temperature range from 220 to 380 K, and reference magnetic properties are given for the same temperature range. Soft magnetic materials with a Curie point near the upper boundary of this interval have the highest magnetic permeability [136], because the magnetic anisotropy constant is lower here. Near the Curie point, the saturation magnetization is also lower and, therefore, there is a tendency to a increase in permeability with decreasing saturation magnetization, see Figure 34b. In non-metallic ferrites, such a connection is absent.

Nanocrystalline soft magnetic materials of the Finemet type have high permeability, up to 100,000, which is achieved at a sufficiently high saturation magnetization, *J_s_* = 1.25 T. A thin ribbon, 20 μm, together with a low coercive *H_c_*, less than 1 A·m^−1^, provide low core losses in a wide frequency range. Magnetic anisotropy, induced in a magnetic field or under stress, allows the formation of a linear hysteresis loop with a low remanence ratio and permeability from 100 to 100,000 or a round hysteresis loop with a high remanence ratio. Nanocrystalline soft magnetic materials are used for frequencies from 50 Hz, for example, for high-precision current transformers, and up to 10 MHz, for example, for attenuation of common-mode noises [137]. The use of nanocrystalline materials in traditional components and devices improves the electromagnetic properties of these products [19] (pp. 277–345), [138].

**Figure 35:**
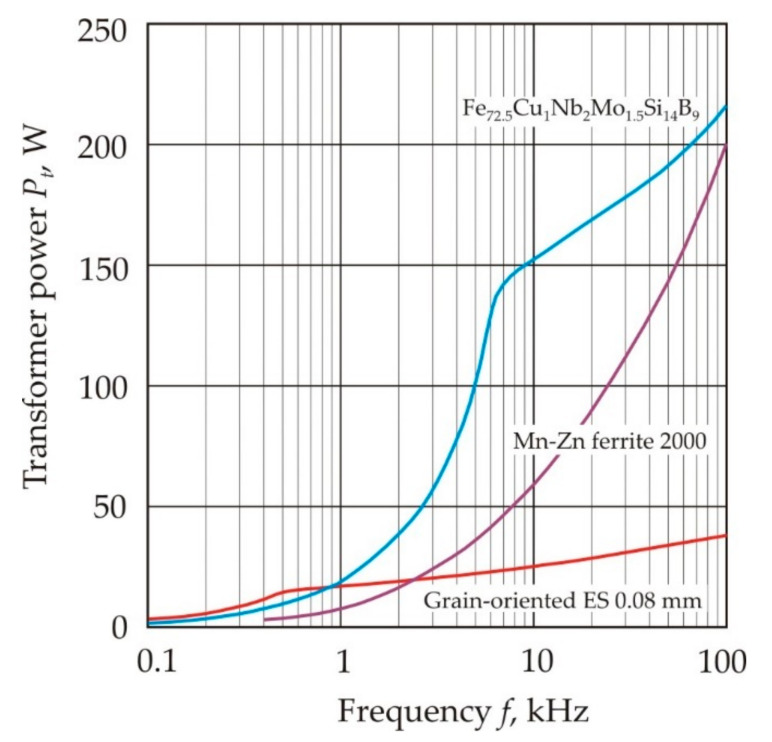
Power of transformers *P_t_* as a function of frequency *f* for a magnetic core made of different soft magnetic materials, which has an outer diameter of 20 mm, an inner diameter of 32 mm and a height of 10 mm. Adapted from [139].

In power electronics, nanocrystalline materials are most commonly used for transformers and electrical reactors or chokes. Figure 35 shows the power of transformers *P_t_* with a magnetic core, which has an outer diameter of 20 mm, an inner diameter of 32 mm and a height of 10 mm [139]. The core was made of grain-oriented electrical steel 0.08 mm thick, MnZn ferrite with permeability 2000, and nanocrystalline Fe_72.5_Cu_1_Nb_2_Mo_1.5_Si_14_B_9_ alloy. The calculation was carried out for a transformer overheating temperature of no more than 20 K, a current density in the windings of 3 × 10^6^ A·m^−2^, and a window fill factor for the wires of the primary and secondary windings of 0.2.

From Figure 35 it follows that up to almost 100 kHz, the power of a nanocrystalline alloy transformer is higher compared to other transformers. An exception is an electrical steel transformer with a frequency of less than 1 kHz. Higher power in such a transformer is provided by a higher saturation magnetization in electrical steel. The power of the nanocrystalline alloy transformer rises sharply to a frequency of about 7 kHz. Such an increase is associated with a continuous increase in the operating induction of the transformer to a limit value of 1.1 T. At an increased frequency, the operating induction has to be reduced in order to meet the overheating temperature requirement. In a ferrite transformer, the maximum operating induction is achieved already at a frequency of less than 1 kHz. In the frequency range from 1 to 10 kHz, nanocrystalline magnetic cores have a significant advantage over other soft magnetic materials.

**Figure 36:**
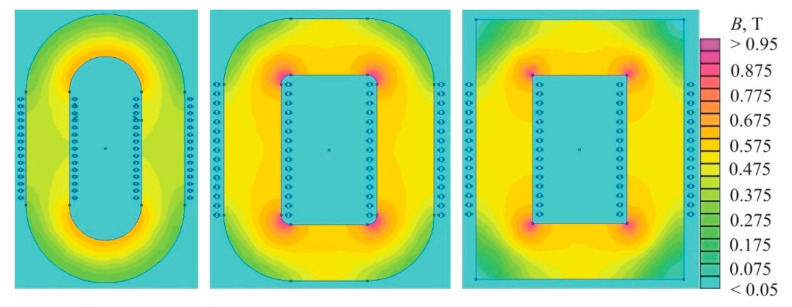
Distribution of magnetic induction *B* in cores made of nanocrystalline Fe_72.5_Cu_1_Nb_2_Mo_1.5_Si_14_B_9_ alloy and having different shapes. Reproduced with permission from [19] (p. 96).

The shape of the magnetic cores greatly affects the distribution of the magnetic flux. Figure 36 shows the distribution of magnetic induction *B* in cores with a cross section of 60 × 125 mm, which are made of nanocrystalline Fe_72.5_Cu_1_Nb_2_Mo_1.5_Si_14_B_9_ alloy [19] (p. 96). The cores had different radius of curvature at the junction of the limb and yoke. The magnetic field was created by a winding of 15 turns on each limb, through which a current of 0.5 A flowed.

Figure 36 shows that the magnetic induction is almost uniformly distributed in the straight limbs [140]. The inhomogeneity of the magnetic induction appears due to the curvature of the magnetic field lines at the junction of the limb and yoke. The inhomogeneity is higher with a smaller radius of curvature. The core with right angles has the greatest inhomogeneity. In the inner areas of the corner, the magnetic induction reaches 1.1 T, while the peripheral areas are almost not magnetized.

**Figure 37:**
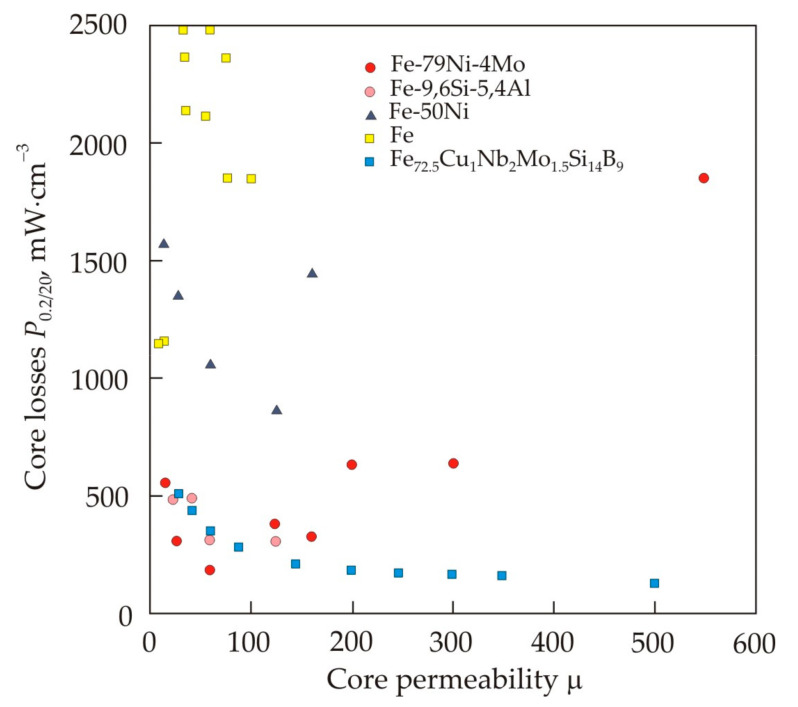
Core losses in magnetodielectric cores and cut cores made of nanocrystalline Fe_72.5_Cu_1_Nb_2_Mo_1.5_Si_14_B_9_ alloy. Adapted from [140].

To store high magnetic energy, the core of an electric reactor (choke) must have a low permeability. In small reactors, cores of magnetodielectric materials are used, which are obtained by pressing magnetic particles isolated from each other. An alternative is the cut magnetic cores. Figure 37 compares the core losses at *f* = 20 kHz and *B_m_* = 0.2 T in magnetodielectrics and in cut cores made of nanocrystalline Fe_72.5_Cu_1_Nb_2_Mo_1.5_Si_14_B_9_ alloy [140]. Magnetodielectrics made of Mo-permalloy, sendust, and Fe_72.5_Cu_1_Nb_2_Mo_1.5_Si_14_B_9_ alloy have approximately the same level of core losses, but only for permeability below 140. High permeability cores are best made from nanocrystalline soft magnetic materials. A significant increase in losses in magnetodielectrics with a permeability above 140 is associated with a decrease in the dielectric properties of the insulation between the magnetic particles.

## 8. Conclusions

The review analyzes the physical processes occurring at different stages of the production and application of nanocrystalline soft magnetic materials. The object of the study was Fe–Si–B alloys doped with various chemical elements, which have found wide practical application.

When a multicomponent melt is heated above a certain critical temperature, the melt viscosity at the cooling stage does not coincide with the viscosity at the heating stage, and the heating and cooling curves can form a hysteresis loop. The critical temperature is associated with the rearrangement of the melt structure, in particular, with the formation of a more uniform structure in the high-temperature region. An amorphous precursor from a homogeneous melt has a higher plasticity and enthalpy of crystallization, and after nanocrystallization, a material with a higher permeability is obtained.

The most effective inhibitor elements have a large atomic size, they are practically insoluble in α-Fe and form a smoothed peak of heat release during crystallization. On the other hand, the finest nanograins and the highest permeability are obtained when the heat release has a narrow high-temperature peak. The role of soluble elements in the formation of magnetic anisotropy induced by the magnetic field and stress is shown.

The cluster magnetic structure of nanocrystalline alloys is manifested in magnetic inhomogeneity. The influence of magnetic inhomogeneity on the shape of the magnetic hysteresis loop and on core losses is shown, and the mechanism of irreversible magnetization rotation is considered.

Nanocrystalline soft magnetic materials based on iron have a whole set of magnetic properties, which made it possible to expand their application in a short time. The nanocrystalline alloys have high saturation magnetization and permeability, low coercive force, magnetostriction and core losses, as well as the ability to change the shape of the hysteresis loop due to magnetic-field-induced or stress-induced anisotropy. Further research in the field of nanocrystalline soft magnetic materials is aimed at developing new efficient and economical nanocrystalline materials, including those with high saturation magnetization for an industrial frequency. An important task remains the development of new magnetic components and electromagnetic devices based on already available nanocrystalline soft magnetic materials.

## Figures and Tables

**Table 1:** Activation energy of viscous flow for Fe_73.5_Cu_1_M_3_Si_13.5_B_9_ melts with different inhibitors M = Nb, Mo, V, Cr. Reproduced with permission from [36].

Inhibitors, M	Activation Energy *E_a_*, kJ·mol^−1^			
	Heating		Cooling	
	*T* < 1770 K	*T* > 1770 K	*T* < 1770 K	*T* > 1770 K
Nb	21	42	35	35
Mo	47	41	41	41
V	43	34	36	36
Cr	38	38	40	40

**Table 2:** The activation energy *E_a_*, cluster size *d*, and pre-exponential factor ν_0_ in the overheated and not overheated melt obtained from the amorphous Fe_72.5_Cu_1_Nb_2_Mo_1.5_Si_14_B_9_ ribbon [45].

Temperature Range	Heating			Cooling		
	*E_a_*, kJ·mol^−1^	ν_0_·10^8^,·m^2^·s^−1^	*d*, nm	*E_a_*, kJ·mol^−1^	ν_0_·10^8^,·m^2^·s^−1^	*d*, nm
	Overheated melt					
1470–1670 K	23.2	26.3	0.05	26.0	21.1	0.05
1670–1870 K	51.4	3.47	2.5	45.7	4.8	1.5
	Not overheated melt					
1470–1670 K	37.6	7.6	0.4	38.2	4.0	1.5
1670–1750 K	83.1	0.27	380	76.1	0.26	410
1750–1870 K	44.1	2.31	6	38.4	3.47	2.5

**Table 3:** Magnetic properties of Fe_73.5_Cu_1_M_3_Si_13.5_B_9_ alloys with different inhibitors after annealing at *T_a_* = 823 K, 1 h. Reproduced with permission from [77].

Inhibitors	Initial Permeability μ_0.08_	Coercive Force *H*_c_, A⋅m^−1^	Core Losses *P*_0.2/20_, W⋅kg^−1^	Disaccommodation *D*_90_
Nb	180,000	0.6	7.0	0.055
W	130,000	0.9	4.5	0.30
Mo	80,000	0.8	5.0	0.30
V	1400	45	-	-
Cr	100	550	-	-
Mo_0.5_Nb_0.5_	140,000	0.6	5.5	0.15
Mo_0.5_W_0.5_	110,000	0.9	4.0	0.35
Mo_0.5_W_0.25_Nb_0.25_	150,000	0.5	4.0	0.15
